# Genome-Based Characterization of Plant-Associated *Rhodococcus qingshengii* RL1 Reveals Stress Tolerance and Plant–Microbe Interaction Traits

**DOI:** 10.3389/fmicb.2021.708605

**Published:** 2021-08-18

**Authors:** Theresa Kuhl, Soumitra Paul Chowdhury, Jenny Uhl, Michael Rothballer

**Affiliations:** ^1^Institute for Network Biology, Helmholtz Zentrum München – German Research Center for Environmental Health (GmbH), Neuherberg, Germany; ^2^Research Unit Analytical Biogeochemistry, Helmholtz Zentrum München – German Research Center for Environmental Health (GmbH), Neuherberg, Germany

**Keywords:** *Rhodococcus qingshengii*, plant–microbe interaction, quorum quenching, mercury tolerance, nitrogen fixation

## Abstract

Stress tolerant, plant-associated bacteria can play an important role in maintaining a functional plant microbiome and protecting plants against various (a)biotic stresses. Members of the stress tolerant genus *Rhodococcus* are frequently found in the plant microbiome. *Rhodococcus qingshengii* RL1 was isolated from *Eruca sativa* and the complete genome was sequenced, annotated and analyzed using different bioinformatic tools. A special focus was laid on functional analyses of stress tolerance and interactions with plants. The genome annotation of RL1 indicated that it contains a repertoire of genes which could enable it to survive under different abiotic stress conditions for e.g., elevated mercury concentrations, to interact with plants via root colonization, to produce phytohormones and siderophores, to fix nitrogen and to interact with bacterial signaling via a LuxR-solo and quorum quenching. Based on the identified genes, functional analyses were performed *in vitro* with RL1 under different growth conditions. The *R. qingshengii* type strain djl6 and a closely related *Rhodococcus erythropolis* BG43 were included in the experiments to find common and distinct traits between the strains. Genome based phylogenetic analysis of 15 available and complete *R. erythropolis* and *R. qingshengii* genome sequences revealed a separation of the *R. erythropolis* clade in two subgroups. First one harbors only *R. erythropolis* strains including the *R. erythropolis* type strain. The second group consisted of the *R. qingshengii* type strain and a mix of *R. qingshengii* and *R. erythropolis* strains indicating that some strains of the second group should be considered for taxonomic re-assignment. However, BG43 was clearly identified as *R. erythropolis* and RL1 clearly as *R. qingshengii* and the strains had most tested traits in common, indicating a close functional overlap of traits between the two species.

## Introduction

Anthropogenic activities over the past decades, including pollution with heavy metals, pesticides and chemical fertilizer, as well as improper soil exploitation coupled with climate change have led to immense global soil degradation (Smith et al., 2016). This has resulted in loss of soil biodiversity, increase in pathogens and has created harsh biotic and abiotic conditions for plants and their associated microbes (Foley et al., 2005; Brevik and Burgess, 2014; Tsiafouli et al., 2015; Banerjee et al., 2019; Wagg et al., 2019). To survive difficult environmental conditions and maintain a functional plant holobiont (stress resistant) beneficial bacteria are important. Over the last decades, several mechanisms have been identified which are involved in beneficial associations between plant and microbes. They either involve plant growth promotion based on production of plant hormones and providing enhanced nutrients to the plants (Glick, 2012) or plant protection against plant pathogens by producing antimicrobial compounds (Chowdhury et al., 2015a,b) or acting indirectly by inducing host systemic resistance (Kloepper and Beauchamp, 1992; Pieterse et al., 2014; Bahramisharif and Rose, 2019). Apart from that, beneficial bacteria have been shown to support plants as stress protecting agents under abiotic stress conditions like salt and drought stress (Alavi et al., 2013; Berg et al., 2013; Kaushal and Wani, 2016; Bhat et al., 2020) or by supporting plant growth in contaminated soils (Sessitsch et al., 2013; Ma et al., 2016a,b). However, not only plant-microbe interactions, but also microbe-microbe interactions influence the functionality of the plant holobiont. For example, it has been shown that members of the genus *Variovorax* play a major role in shaping the microbiome and with this influence root growth in *Arabidopsis* by balancing auxin production (Finkel et al., 2020). This report emphasizes the importance to understand the role of all relevant members in the plant holobiont. The multifaceted interactions of plants and several plant-associated bacteria have been widely studied to understand the underlying molecular mechanisms and to exploit plant beneficial traits for sustainable agriculture (Berg et al., 2017; Rodriguez et al., 2019; Babin et al., 2021; Windisch et al., 2021). In this context it is important to further the knowledge of the functional repertoire also of yet lesser known members of the plant microbiome such as *Rhodococcus* to understand what enables them to interact with the plant as well as other microbes and which traits could be useful for an application in specifically demanding agricultural scenarios.

Members of the genus *Rhodococcus* are resistant to various stresses (Dabrock et al., 1994; Weyens et al., 2013; Pátek et al., 2021) and are able to degrade and metabolize a large spectrum of toxic compounds (Kamble et al., 2013; Lincoln et al., 2015; Pham et al., 2015; Ceniceros et al., 2017; Gupta et al., 2019; Gorbunova et al., 2020). These traits make the genus *Rhodococcus* interesting for bioremediation applications (Leigh et al., 2006; Płociniczak et al., 2017). Moreover, in metagenomic and microbiome analyses the genus *Rhodococcus* has been frequently reported as an established member of the plant microbiome (Francis and Vereecke, 2019; Vereecke et al., 2020). Many plant associated *Rhodococci* also show plant beneficial traits *in vitro* (Trivedi et al., 2007; Abbamondi et al., 2016; Murugappan et al., 2017) and *in planta* (Belimov et al., 2001).

The here studied closely plant associated *Rhodococcus qingshengii* RL1 was isolated from surface sterilized Rucola (*Eruca sativa* L.) leaves and the genome was recently sequenced (Kuhl et al., 2019). The type strain of species *R. qingshengii* djl-6 was isolated from a carbendazim polluted soil (Xu et al., 2007). Other *R. qingshengii* isolates have been shown to possess nitrogen fixing capacity improving growth of chick pea plants (Joshi et al., 2019) and to produce high amounts of IAA *in vitro* (Hasuty et al., 2018). In combination with the genetic traits for bioremediation, stress resistance, and biocontrol on plants widely distributed across the whole *Rhodococcus* genus these reports clearly warrant a characterization of our new isolate *R. qingshengii* RL1.

We were able to assess the genomic potential of RL1 by characterizing beneficial traits in the genome (Levy et al., 2018) and found that RL1 is well equipped with genes essential for survival under different abiotic stress conditions and for microbial interactions with other microbes and plants. *In vitro* assays were used to assess if these genomic potential was actually transferred into functional traits. To our knowledge, this is the first report of a *R. qingshengii*, which survives under mercury stress and degrades quorum quenching signals (AHLs). Additionally, for the first time we could identify a potential gibberellin producing operon in an actinobacterial genus as well as indications for existence of alternative nitrogen fixation pathways. For establishment of the taxonomic position of RL1 we performed a whole genome based phylogenetic analysis of 15 available and complete *R. erythropolis* and *R. qingshengii* genome sequences and included the *R. qingshengii* type strain djl6 and a closely related *R. erythropolis* BG43 in the *in vitro* experiments. Thus, this work contributes to the elucidation of molecular mechanisms and underlying genetic determinants of plant–microbe interactions and possible functions in the plant holobiont of *R. qingshengii* RL1 and closely related strains.

## Materials and Methods

### Bacterial Strains and Growth Conditions

*Rhodococcus qingshengii* RL1, named hereafter RL1, is a gram-positive Actinobacterium and was isolated from Rucola (*Eruca sativa* L.) leaves. Colonies appear in off-white, beige colors. The overnight grown culture corresponds with OD_600_ = 0.42 representing approximately 4 × 10^7^ CFU (colony forming units). *Rhodococcus qingshengii* djl6 DSM 45222 (type strain), named hereafter djl6 (Xu et al., 2007) and *Rhodococcus erythropolis* BG43 DSM 46869, named hereafter BG43 (Müller et al., 2014) were obtained from German Collection of Microorganisms and Cell Cultures (DSMZ, Braunschweig). The overnight grown cultures correspond with OD_600_ = 0.5 representing approximately 5 × 10^7^ CFU and OD_600_ = 0.92 representing approximately 5 × 10^8^ CFU, respectively. *Rhodococcus* strains were cultivated in tryptic soy broth (TSB, Sigma, United States) [casein peptone (pancreatic), 17 g/L, dipotassium hydrogen phosphate, 2.5 g/L, glucose, 2.5 g/L, sodium chloride, 5 g/L, soya peptone (papain digest.), 3 g/L] or solid tryptic soy agar (1.7% agar) with pH 7.3, unless further specified, at 28°C and 180 rpm.

Control strains for the conducted experiments were: the strain *Bacillus velezensis* FZB42 DSM 23117, producing fungal antagonistic compounds like surfactin, fengycin and iturin (Chowdhury et al., 2015a), the AHL biosensor strain *Agrobacterium tumefaciens* A136 ATCC 51350 (Stickler et al., 1998; Han et al., 2016), the AHL producer strain *Acidovorax radicis* N35 DSM 23535 (Li et al., 2011) the non-AHL-producing mutant strain *Acidovorax radicis* N35 AHL- *araI::tet* (Han et al., 2016), the AHL-degrading mutant strain *Rhizobium radiobacter* F4 AHL- expressing an AHL lactonase (AiiA) (Alabid et al., 2020) and able to grow on potassium tellurite trihydrate (K2TeO_3_^∗^3H_2_O) 100 μg/ml, the phosphate solubilizing strain *Luteibacter* sp. Cha2324a_16 and the ACC utilizing strain *Variovorax* sp. M92526_27 isolated from wheat roots (this study), the nitrogen-fixing and IAA producing *Herbaspirillum frisingense* GSF30 DSM 1328 (Kirchhof et al., 2001), the nitrogen-fixing *Azospirillum brasilense* Sp7 DSM 1690 (Tarrand et al., 1978; Hartmann and Hurek, 1988), the biofilm-producing *Pseudomonas simiae* WCS417r (Pieterse et al., 2020) and the non-biofilm-producing *Escherichia coli* DH5α (Anton and Raleigh, 2016). Strains were cultivated in liquid nutrient broth (NB, Roth, Germany) (beef extract 3 g/l, gelatin peptone 5 g/l) or solid nutrient agar (with 1.7% agar) with pH 6.8, unless further specified, at 28°C at 180 rpm.

### Genome Comparison

The genome of *Rhodococcus qingshengii* RL1 (Kuhl et al., 2019) was compared to the genomes of the type strain *Rhodococcus qingshengii* djl6 (Xu et al., 2007; Wang et al., 2010; Táncsics et al., 2014), as well as the closely related soil isolate *Rhodococcus erythropolis* BG43 (Rückert et al., 2015). The genome djl6 is based on the species *R. jialingiae* (Wang et al., 2010) which was later identified as a synonym of the type strain *R. qingshengii* (Táncsics et al., 2014). For the genome comparison and the identification of orthologous and unique genes in the three different genomes the efficient database framework for comparative Genome Analyses using BLAST score Ratios – EDGAR (Blom et al., 2016) was used.

### Functional Annotation of RL1 Genome

The RL1 genome was annotated upon submission to NCBI with the NCBI prokaryotic Genome Annotation Pipeline (PGAP) with the annotation method best-placed reference protein set with GeneMarkS-2+ and the Rapid Annotation using Subsystem Technology 2.0 (RAST) with default parameter of the classicRAST annotation scheme plus frameshift fixing and backfilling of gaps allowed, where the annotated genome was browsed afterward in the SEED environment (Aziz et al., 2008; Overbeek et al., 2014). Functional annotation by grouping genes in clusters of orthologous groups (COG) of proteins according to Tatusov et al. (2000) was performed with eggNOG v5.0 (Jensen et al., 2008). Genes were annotated with the KEGG (Kyoto Encyclopedia of Genes and Genomes) orthology (KO) identifiers, or the K numbers, and directly linked to the KEGG pathways with the KEGG automatic annotation server (KAAS) (Moriya et al., 2007) and KEGG Mapper. Further functional annotation was performed by identifying plant microbe interaction factors and gene clusters for biosynthesis of secondary metabolites with Plant–bacteria Interaction Factors Resource (PIFAR) (Martínez-García et al., 2016) and the antibiotics and secondary metabolite analysis shell – antiSMASH (Blin et al., 2017) using the default parameters.

### Phylogenetic Analysis

Sixty-one complete genomes of the genus *Rhodococcus* and the genome of the out-group *Streptomyces albus* NBRC 13014 (type strain), were used for the full-genome approximately maximum-likelihood phylogenetic tree build in EDGAR. 15 genomes identified in the phylogenetic tree as members of the *Rhodococcus erythropolis* clade and two out-group genomes were used for the approximately maximum-likelihood phylogenetic tree calculated in EDGAR using FastTree Software with the Shimodaira–Hasegawa test for bootstrap values. Average nucleotide identity (ANI) and Average amino acid identity (AAI) was calculated in EDGAR (Blom et al., 2016) as described in Konstantinidis and Tiedje (2005) and Goris et al. (2007).

### Evaluation of Growth and Tolerance to Different Stress Factors

If not indicated otherwise all *Rhodococcus* strains were pre-cultured in liquid TSB overnight.

#### Mercury Tolerance

Overnight grown cultures were transferred to fresh TSB medium with increasing mercury levels 0.001, 0.01, 0.1, and 1 mM adjusted with mercury-II-chloride (HgCl_2_, Roth, Germany) according to Dziewit et al. (2013) and incubated at 28°C. Growth rates were evaluated by spectrophotometric measurement of optical density at 600 nm (OD_600_) after 24 and 48 h. For treatments without detectable growth (=0.1 mM and 1 mM mercury), the recovery of cells was evaluated by the ability to form colonies on TSB agar plates without mercury. Hundred microliter of cultures from the treatments with 0.1 and 1 mM mercury were plated on TSB without mercury and incubated at 28°C for 24 and 48 h. Experiment was repeated three times. Non-mercury-tolerant strains *Herbaspirillum frisingense* GSF30 and *Bacillus velezensis* FZB42 served as negative controls.

#### Salt Stress Tolerance

Overnight grown cultures were transferred to fresh TSB medium with increasing sodium chloride (NaCl, Merck, Germany) levels 0, 1, 2.5, 3.5, 5.5, 7.5, 12, and 15% according to De Carvalho et al. (2014) and incubated at 28°C. Growth rates were evaluated by spectrophotometric measurement of optical density at 600 nm (OD_600_) after 24 and 48 h. For treatments without detectable growth (12 and 15% NaCl), the recovery of cells was evaluated by the ability to form colonies on TSB agar plates without NaCl. Hundred microliter of cultures from the treatments 12 and 15% were plated on TSB agar without NaCl and incubated at 28°C for 24 and 48 h. Experiment was repeated three times. Less salt tolerant *Herbaspirillum frisingense* GSF30 and *Bacillus velezensis* FZB42 served as negative controls.

#### pH Tolerance

Overnight grown cultures were transferred to fresh TSB medium with pH values 8, 7, 6, 5, 4, 3, 2 adjusted with hydrochloric acid (HCl, Merck, Germany) or sodium hydroxide (NaOH, Sigma, United States) and incubated at 28°C. Growth rates were evaluated by spectrophotometric measurement optical density at 600 nm (OD_600_) after 24 and 48 h. Recovery was evaluated by the ability to form colonies on TSB agar plates at pH 7.3 after 48 h in treatments without detectable growth (pH 4, 3 and 2). Hundred microliter of medium from the treatments pH 4, 3, and 2 were plated on TSB and incubated at 28°C for 24 and 48 h. The experiment was repeated three times. *Herbaspirillum frisingense* GSF30 and *Bacillus velezensis* FZB42 which did not grow in low pH (below 5) served as negative controls.

#### Osmotic Stress Tolerance

Overnight grown cultures were transferred to fresh TSB medium with increasing osmotic stress levels 0, –0.25, –0.5, –0.75, –1, –1.25, and –1.5 MPa and incubated at 28°C. Increasing osmotic stress was adjusted with polyethylene glycol 6000 (PEG6000, Serva Electrophoresis GmbH, Heidelberg, Germany) based on decreasing water potential with the formula of Kaufmann and Michel (1973), according to Kumar et al. (2014) and Jayakumar et al. (2020). –1.5 MPa is the water potential plants in regular soil start to wilt irreversibly. Growth rates were evaluated by spectrophotometric measurement optical density at 600 nm (OD_600_) after 24 and 48 h. Experiment was repeated three times. Gram-negative *Herbaspirillum frisingense* GSF30 and gram-positive *Bacillus velezensis* FZB42 served as controls.

#### Antibiotic Resistance

Overnight grown cultures were diluted 1:10 with fresh TSB medium. Two hundred microliter of the diluted overnight cultures were spread on TSB agar plates and antimicrobial susceptibility test stripes (Himedia Laboratories, India) for kanamycin (0.016–256 μg/ml), ampicillin (0.016–256 μg/ml), rifampicin (0.002–32 μg/ml), and vancomycin (0.016–256 μg/ml) were placed according to manufacturer’s protocol. The inhibition zone was evaluated after 24 and 48 h.

Bacterial strains were streaked on a fresh TSB or NB plate from glycerol stocks and grown overnight. A single colony of RL1 was picked and streaked on nutrient broth (NB) agar plates with 100 μg/ml potassium tellurite trihydrate (K_2_TeO_3_^∗^ 3H_2_O, Sigma, United States) for 48 h. Dark gray colony growth was evaluated as positive growth. The strain *Rhizobium radiobacter* F4 AHL- aiiA- genetically modified to tolerate a tellurite concentration of 100 μg/ml served as positive control.

### Characterization of Traits Involved in Microbe–Plant Interactions

If not indicated otherwise all *Rhodococcus* strains were pre-cultured in liquid TSB overnight.

#### Indole-Acetic Acid Production

Indole-acetic acid (IAA) production was determined by the colorimetric method of Gordon and Weber (1951). Overnight grown cultures were transferred to fresh TSB medium with and without the IAA precursor 5 mM tryptophan (1 mg/mL, Sigma) and grown for 48 h. Liquid cultures were centrifuged for 2 min at 5000 × *g*. Hundred microliter of supernatant were mixed with 100 μl of Salkowski reagent [0.01 M FeCl_3_ anhydrous (Fluka Biochemika, Germany) in perchloric acid (HClO_4_) 35% (Merck, Germany)] (Loper and Schroth, 1986) and 1 μl of orthophosphoric acid (Sigma, United States). After incubation in the dark for 30 min amounts of IAA in the supernatant were analyzed in a plate reader (Spectra Max iD3, Molecular Devices) at 530 nm wavelength. A standard curve was prepared from commercial indole-3-acetic acid (Fluka Biochemika, Germany) in TSB with concentrations ranging from 0 to 100 μg/ml and *Herbaspirillum frisingense* GSF30 was used as positive control. Supernatant measurements were performed in triplicates. Evaluation was based on the amount of produced IAA normalized to an OD_600_ = 1.

#### Siderophore Production

Siderophore production was analyzed according to Pérez-Miranda et al. (2007) and Lynne et al. (2011) with modifications. Twenty-five microliter of overnight grown cultures were spotted on TSB agar plate and grown for 48 h. Dye solutions [chrome azurol blue S (Sigma, United States), FeCl_3_ (Fluka Biochemika, Germany), HDTMA (Hexadecyltrimethylammonium bromide, Sigma, United States)] were prepared and mixed according to Lynne et al. (2011). Piperazin-*N,N’*-bis-(2-ethanesulfonic acid) (Pipes, Roth, Germany) was added to H_2_O with 0.9 % agar and pH was adjusted to 6.8. After autoclaving separately, the dye solution was slowly mixed with the Pipes-Agar mix. Cooled but still liquid overlay agar (10 ml) was poured on plates with bacteria. After 2 h siderophore production was analyzed by detection of color change from blue to orange. The experiment was repeated three times.

#### Phosphate Solubilization

Overnight grown cultures were washed twice in 1x PBS and 25 μl were spotted on National Botanical Research Institute’s phosphate growth medium (NBRIP) according to Nautiyal (1999) and incubated at 28°C. After 6 days, phosphate solubilization activity was determined according to the formation of a clear halo surrounding the spotted colony using the Phosphate Solubilization Index (SI): (Colony diameter + Halo zone diameter)/colony diameter). The phosphate-solubilizing *Luteibacter* sp. Cha2324a_16 served as positive control. The experiment was repeated three times.

#### 1-Aminocyclopropane-1-Carboxylate Utilization

1-Aminocyclopropane-1-carboxylate (ACC, Biozol Diagnostica GmbH, Germany) utilization as nitrogen source was analyzed with M9 minimal medium [Na_2_HPO_4_ 33.1 mM, KH_2_PO_4_ 22 mM, NaCl 8.55 mM (NH_4_Cl 9.35 mM), glucose 0.4%, MgSO_4_ 1 mM, CaCl_2_ 0.3 mM] containing NH_4_Cl 9.35 mM (Roth, Germany) or ACC 3 mM as nitrogen source or no nitrogen source. Overnight grown cultures were washed twice in 1x PBS and 25 μl were spotted on each plate. After 10 days, ACC utilization as nitrogen source was analyzed by comparing bacterial growth on M9, M9 with ACC and nitrogen-free M9 plates. ACC utilizing *Variovorax* sp. M92526_27 served as positive control. The experiment was repeated three times.

#### Nitrogen Fixation

Nitrogen fixation was analyzed with nitrogen-free semi-solid Nfb-medium according to Döbereiner (1995), on Ashby’s mannitol medium (Mannitol 20 g/l, K_2_HPO_4_ 0.2 g/l, MgSO_4_^∗^5H_2_O 0.2 g/l, NaCl 0.2 g/l, K_2_SO_4_ 0.1 g/l, CaCO_3_ 5 g/l, Agar 15 g/l) and on Jensen’s medium (Sucrose 20 g/l, K_2_HPO_4_ 1 g/l, MgSO_4_^∗^5H_2_O 0.5 g/l, NaCl 0.5 g/l, FeSO_4_ 0.1 g/l, Na_2_MoO_4_ 0.005 g/l, CaCO_3_ 2 g/l, Agar 15 g/l). Overnight grown cultures were washed twice in 1x PBS and 10 μl were spotted on nitrogen-free semi-solid Nfb-medium and incubated at 28°C. Pellicle formation was evaluated after 48 h. Ten microliter of washed overnight cultures were streaked on Ashby’s mannitol agar. Bacterial strains were streaked on a fresh TSB or NB plate from glycerol stocks and grown overnight. A single colony of each strain was picked and streaked on Jensen’s agar. Bacteria on Ashby’s medium and Jensen’s medium were incubated at 28°C and growth was evaluated after 3 days. The experiments were repeated three times. Nitrogen-fixing *Azospirillum brasilense* Sp7 served as positive control.

#### Biofilm Formation

Biofilm formation was analyzed according to O’Toole (2011). Overnight grown cultures were washed in 1xPBS and OD_600_ was adjusted to 0.1. Bacterial strains were cultivated in a microtiter plate in 100 μl modified M9 minimal medium with 0.5% casamino acids (Biozol Diagnostica Vertrieb GmbH, Germany) without shaking at 28°C. After incubation OD_600_ was measured in the plate reader (SpectraMax iD3, Molecular Devices). After 24 h OD_600_ was measured and unattached cells were dumped out of the plate. The plate was washed twice by submerging it in MilliQ water to further remove unattached cells. Hundred and twenty-five microliter of 0.1% crystal violet (Roth, Germany) solution was added to each well. After 15 min the plate was rinsed three times in MilliQ water and dried for 1.5 h before visual inspection of biofilm production. For quantification of the biofilm 125 μl of 30% acetic acid (Roth, Germany) was added to each well and incubated for 15 min at room temperature. The solution was transferred to a new microtiter plate and color intensity was quantified at the plate reader (SpectraMax iD3, Molecular Devices) with absorbance at 550 nm and 30% acetic acid as blank. Biofilm-forming *Pseudomonas simiae* WCS417 served as positive control and non-biofilm-producing *Escherichia coli* DH5α served as negative control. The experiment was repeated three times with 6–12 replicates per strain.

### Interactions With Other Organisms

#### Confrontation Assay Against Plant–Pathogenic Fungi

The interaction of RL1 with the well-known plant pathogenic fungi *Rhizoctonia solani*, *Fusarium culmorum*, and *Fusarium oxysporum* was investigated with an *in vitro* confrontation assay. The following pathogenic fungi were used: *Rhizoctonia solani*, causing potato stem cancer and black scurf (Yang and Li, 2012), wheat pathogenic fungus *Fusarium culmorum* G2191 causing seedling blight, foot rot, and head blight (Wagacha and Muthomi, 2007) and the wilt-causing *Fusarium oxysporum* DSM62297 (Gerlach et al., 1958). Fungi were cultivated on potato dextrose agar (PDA, Sigma, United States) (potato extract 4.0 g/L, glucose 20.0 g/L) at room temperature in the dark and stored at 4°C until further use.

RL1 was pre-grown in TSB. Overnight grown culture was diluted to OD_600_ = 0.1 with fresh TSB medium and 10 μl were dripped on the plate. Approximately 1 mm^3^ PDA pieces grown with fungi were aseptically transferred to TSB plates at a distance of approximately 3 cm. After 9 days of growth the zone of inhibition formation was visually analyzed and documented photographically. Sterile water served as negative control and *Bacillus velezensis* FZB42, a known fungal antagonistic strain served as positive control. Confrontation assays were performed in triplicates.

#### Degradation of Synthetic and Bacterial *N*-Acyl-Homoserine Lactones (AHLs)

The identified *qsdA* gene sequence of the RL1 genome encoding the AHL lactonase was used to construct a phylogenetic tree with nearest relatives with MEGA X (Kumar et al., 2018). *Rhodococcus* strains were analyzed with a well diffusion agar-plate assay (Rodríguez et al., 2020) and a V-shaped assay (Berendsen et al., 2018) with modifications.

##### Well diffusion plate assay

For the experiments with synthetic AHL *Rhodococcus* strains were pre-grown in TSB. Overnight grown cultures were transferred to fresh TSB liquid medium supplemented with 10 μM C12-HSL (Biomol GmbH, Germany) and incubated at 28°C 180 rpm. Cell-free TSB medium supplemented with 10 μM C12-HSL served as control. For the co-cultivation experiment RL1 and *Acidovorax radicis* N35e overnight cultures were adjusted to OD_600_ = 0.2 and co-cultured in fresh liquid NB medium. Pure culture of *Acidovorax radicis* N35e served as control.

The well diffusion plates were prepared as follows: The AHL biosensor strain *Agrobacterium tumefaciens* A136 was pre-grown in NB. NB plates were overlaid with soft NB agar (0.5% agar) supplemented with the biosensor strain A136 and 80 μg/ml 5-bromo-4-chloro-3-indolyl-β-D-galactopyranoside (X-gal, Life Technologies GmbH, Germany). Twenty microliter of each supernatant from the co-cultivation or synthetic AHL experiment were filled in wells prepared in the soft agar and incubated at 28°C for 30 h. Remaining AHLs were detected by color change. Pure NB was used as negative control for presence of AHLs.

##### V-shaped plate assay

The AHL biosensor strain *Agrobacterium tumefaciens* A136 was pre-grown in NB. *Agrobacterium tumefaciens* A136 and 80 μg/ml 5-bromo-4-chloro-3-indolyl-β-D-galactopyranoside (X-gal, Life Technologies GmbH, Germany) were spread on NB plates. *Rhodococcus* strains were pre-grown in TSB. The AHL producing strains *Acidovorax radicis* N35e was pre-grown in NB, non-AHL-producing mutant strain *Acidovorax radicis* N35 AHL- *araI::tet* was pre-grown in NB with tetracyclin 20 μg/ml and kanamycin 50 μg/ml. Overnight grown cultures were washed in 1x PBS and optical density was adjusted to OD_600_ = 0.1. Eight times 1 μl of each culture was dripped in a diagonal row on the prepared NB plates in V-shape with increasingly closer inoculation sites. Plates were incubated for 30 h at 28°C. AHL degradation was detected by color change.

### Evaluation of Rhizosphere Competence

#### Root Inoculation in Axenic System

Rucola (*Eruca sativa* L.) seeds were washed in Tween 80 1% (Sigma, United States) for 2 min, surface sterilized with sodium hypochlorite 12% (NaOCl, Roth, Germany) for 8 min and washed three times in sterile deionized water for 2 min. Sterilized seeds were placed on Hoagland’s solution (Sigma, United States) with 0.8% agar to germinate 4 days. *Rhodococcus* strains were pre-grown in TSB. Overnight grown cultures were washed two times in 1x PBS (AppliChem, Germany) and diluted to a concentration of 10^7^ CFUs. Seedlings were inoculated in the prepared bacterial solution of RL1, BG43, and djl6 for 1 h under shaking at 160 rpm at 28°C. Seedlings inoculated in 1x PBS served as negative control. Inoculated seedlings were transferred to an axenic system with 80 ml sterile quartz sand and 20 ml Hoagland’s solution in a sterile Phytatray II (Sigma, United States). Seedlings inoculated with RL1 were additionally transferred on plates with 0.5x Murashige and Skoog Medium (0.5x MS) including vitamins (Duchefa Biochemie, Netherlands); pH was adjusted to 5.7 with 2N KOH. No additional sucrose was added to 0.5x MS. The axenic system was placed in a Phytochamber (Weiss Technik, Modell SGC120PG2, Germany) with 23°C, 55% humidity, day-night-cycle 12 h : 12 h. After seven and 14 days freshly harvested roots were washed in 1x PBS, fixed in 55% EtOH and 1x PBS mix and stored at –20°C until further use.

#### Fluorescence *in situ* Hybridization (FISH)

Fluorescence *in situ* hybridization was performed following the protocol of Alquéres et al. (2013). Chemicals were obtained from AppliChem, Germany. After an increasing ethanol series [(50, 80, and 96% [vol/vol] for 3 min each] for fixation and desiccation, roots were incubated in 50 μl hybridization buffer [0.9 M NaCl, 0.01% sodium dodecyl sulfate (SDS), 10 mM Tris-HCl (pH 8.0), 35% deionized formamide] with 15 pmol of the fluorescently labeled probes EUB338, specific for eubacteria (Amann et al., 1990; Daims et al., 1999) and labeled with fluorescein (FITC, Thermo Scientific, Germany), and HGC69a (Roller et al., 1994), specific for bacteria with high G + C content in their 16S rRNA and labeled with Cy3 (Thermo Scientific, Germany) or ATTO550. Hybridization was performed for 1.5 h at 46°C.

#### Confocal Laser Scanning Microscopy (CLSM)

FISH stained roots and bacterial cells were investigated at the Zeiss confocal laser scanning microscope LSM880 (Zeiss, Oberkochen, Germany) with argon ion laser and helium neon laser for excitation of FITC (488 nm), Cy3 (561 nm) and an unlabeled control channel (633 nm). Cells were observed with a 64x C-Apochromat water immersion objective. Micrographs were recorded using the software Zen Black Edition (Zeiss, Oberkochen, Germany).

#### Quantitative Evaluation

Rhizosphere competence of the investigated bacterial strains were estimated via counting of colony forming units (CFU). Sterilized Rucola (*Eruca sativa* L.) seedlings were inoculated in bacterial solution and planted in the axenic system as described above. After 7 days three roots per treatment were harvested, weighed and ground in a sterilized mortar with 1 ml 1x PBS. Ground roots were diluted three times (10^–3^), 100 μl of each dilution was plated in triplicates on TSB plates and incubated at 28°C. After 48 h CFUs of dilution 10^–3^ were counted and mean values were compared between treatments. Plating of dilutions 10^–1^ and 10^–2^ resulted in too many CFUs for counting.

### Statistical Analysis

Sample size was not predetermined using statistical methods. Statistical analysis was performed with RStudio 3.6.1. Data were tested for normal distribution with Shapiro–Wilk-Test and analyzed with the non-parametric Fligner-Killeen-Test or with analysis of variances (ANOVA) followed by the post-hoc analysis with Tukey’s test. Significance level was 5% marked in the graphs by asterisks.

## Results

### Phylogenetic Analysis

**FIGURE 1:**
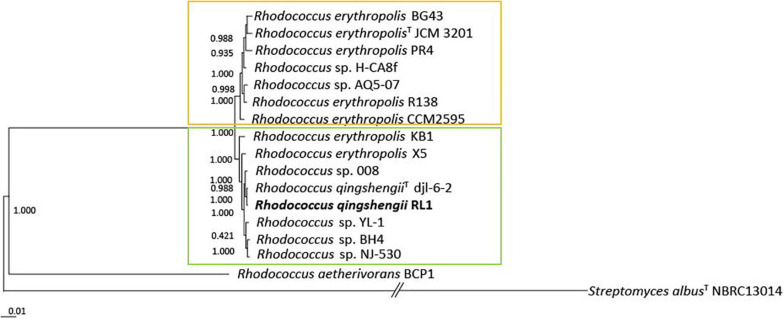
Maximum-likelihood phylogenetic tree of 15 genomes of the *Rhodococcus erythropolis* clade generated with FastTree 2.1 from 1211 nucleotide sequences of the core genome. Values represent local support values based on Shimodaira–Hasegawa test (1 SH = 100% bootstrap). The scale bar represents nucleotide substitutions per site (0.01 scale = 1% nucleotide substitutions per site). The *Rhodococcus erythropolis* subgroup is marked in orange and the *Rhodococcus qingshengii* subgroup in green.

The full genome based phylogenetic tree of *Rhodococcus* was constructed on a core genome of 633 genes from 39246 genes in total (Supplementary Figure 1). Based on this phylogenetic tree 15 genomes of the *R. erythropolis* clade were chosen to calculate the full genome based phylogenetic tree of the *R. erythropolis* clade. It was built on a core genome of 1211 genes from 20587 genes in total and revealed that the clade can be separated into two groups (Figure 1). The first group includes *R. erythropolis* strains only. The second group harbors a mix of *R. erythropolis* and *R. qingshengii* strains. ANI values between all analyzed *R. erythropolis* or *R. qingshengii* genomes were higher than 94% (Supplementary Figure 2). The ANI value within the first group was 98.02–98.8% and within the second group 97.17–99.3%. The outgroups *Rhodococcus aethiovorans* and *Streptomyces albus* had ANI values of 72.13–72.57% and 66.79–67.9%, compared to the first group and the second group, respectively. AAI values between the first and the second group of the *R. erythropolis* clade were all above 98% (Supplementary Figure 3), and between both groups and the outgroups *R. aethiovorans* and *S. albus* AAI values were 56.45–57.33% and 76.71–76.81, respectively. *R. qingshengii* djl6 and RL1 grouped together in the second group. *R. erythropolis* BG43 was allocated to the first group.

**FIGURE 2:**
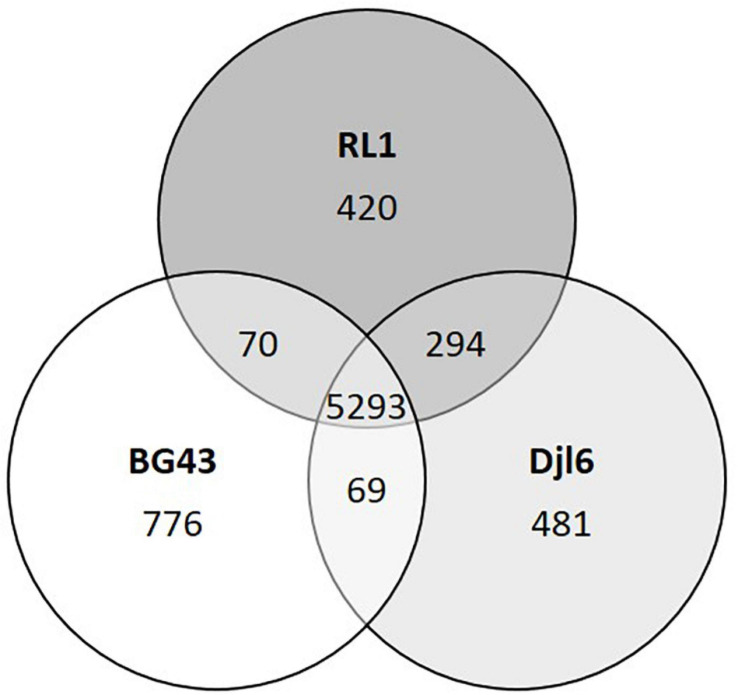
Venn-diagram representing comparative distribution of shared genes between the genomes of RL1, djl6, and BG43. Data obtained with EDGAR Software. Unique genes or singletons are here genes without any hit (BLAST) against any other genome.

Comparing the RL1 genome with the genomes of *R. qingshengii* djl6 and *R. erythropolis* BG43, 5293 genes could be identified that were shared between all three strains (Figure 2). RL1 and djl6 shared more genes (294) than each of them with BG43 (69; 70). For RL1 39 singleton genes could be identified of which 17 were annotated as hypothetical proteins (Table 1).

**TABLE 1:** Singleton genes RL1 based on genome comparison with BG43 and djl6 in EDGAR.

Category	Annotated gene	Gene locus tag RL1
Aromatic carbon metabolism	Enoyl-CoA hydratase/isomerase family protein	D6M20_RS19280
	Fumarylacetoacetate hydrolase	D6M20_RS19325
pH tolerance	Squalene cyclase	D6M20_RS23450
DNA phosphorothioation	Cysteine desulfurase *DndA*	D6M20_RS05845
	DNA sulfur modification protein *DndB*	D6M20_RS05850
	DNA phosphorothioation system sulfutransferase *DndC*	D6M20_RS05855
	DNA sulfur modification protein *DndD*	D6M20_RS05860
	*DndE*	D6M20_RS05865
	DNA phosphorothioation-associated putative methyltransferase	D6M20_RS05870
	DNA phosphorothioation-associated protein 4	D6M20_RS05875
Multidrug resistance	DAED/DEAH box helicase family protein	D6M20_RS05885
	GIY-YIG nuclease family protein	D6M20_RS05895
	Plasmid pRiA4b ORF-3 family protein	D6M20_RS05905
Heavy metal resistance	IS110 family transposase	D6M20_RS28370
	Alkylmercury lyase	D6M20_RS28375
	*merR* family DNA-binding protein	D6M20_RS28380
Other	AAA family ATPase	D6M20_RS05880
	Bifunctional 3-(3-hydroxy-phenyl)propionate/3-hydroxycinnamic acid hydroxylase	D6M20_RS19290
	MFS transporter	D6M20_RS19300
	Helix-turn-helix domain-containing protein	D6M20_RS19305
	DUF3500 domain-containing protein	D6M20_RS19315
	FCD domain-containing protein	D6M20_RS19320
Hypothetical proteins		D6M20_RS05890, D6M20_RS06865, D6M20_RS09680, D6M20_RS09985, D6M20_RS10445, D6M20_RS17155, D6M20_RS17400, D6M20_RS17770, D6M20_RS17780, D6M20_RS18200, D6M20_RS19225, D6M20_RS19295, D6M20_RS19310, D6M20_RS21385, D6M20_RS23515, D6M20_RS23590, D6M20_RS28335

### Functional Annotation of RL1 Genome

**FIGURE 3:**
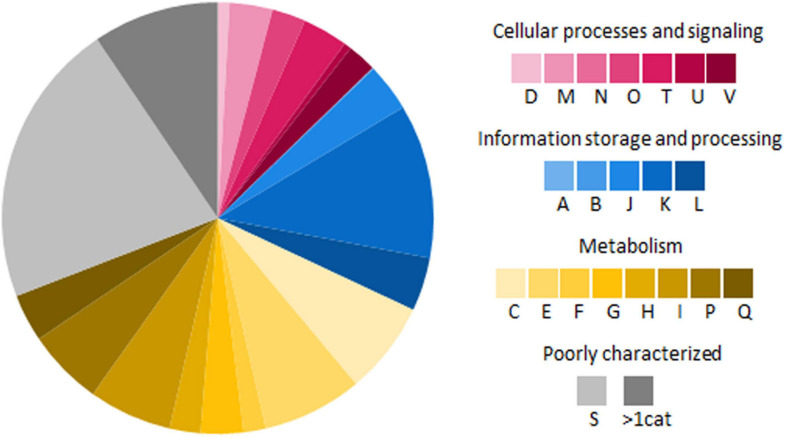
Functional classification of genes encoding proteins in RL1 based on cluster of orthologous groups (COG) (Tatusov et al., 2000). All alphabets represent different COG functional classes: **A**, RNA processing and modification; **B**, Chromatin structure and dynamics; **C**, energy production and conversion; **D**, cell cycle control, cell division, and chromosome partitioning; **E**, amino acid transport and metabolism; **F**, nucleotide transport and metabolism; **G**, carbohydrate transport and metabolism; **H**, coenzyme transport and metabolism; **I**, lipid transport and metabolism; **J**, translation, ribosomal structure, and biogenesis; **K**, transcription; **L**, replication, recombination, and repair; **M**, cell wall, cell membrane, and cell envelope biogenesis; **N**, cell motility; **O**, posttranslational modification, protein turnover, and chaperones; **P**, inorganic ion transport and metabolism; **Q**, secondary metabolites biosynthesis, transport, and catabolism; **S**, no functional prediction; **T**, signal transduction mechanisms; **U**, intracellular trafficking, secretion, and vesicular transport; and **V**, defense mechanisms; **>1 cat**, classified in more than 1 category.

A total of 6,554 protein coding sequences were predicted from the genome of RL1 with (RAST) and 6,328 genes with PGAP (Table 2). 5918 of the predicted genes could be annotated to an assigned function and 92.4% of them were classified into 21 clusters of orthologous groups (COG) identified with eggNOG (Figure 3). Genes involved in metabolism represented the largest fraction (37.1%), followed by information and storage processing (19.2%), and cellular processes and signaling (12.7%) (Figure 3). In more details, the highest number of genes could be assigned to be involved in transcription (K, 11.5%), followed by amino acid transport and metabolism (E, 7.5%) and energy production and conversion (C, 6.9%). 3.6% of the genes could be assigned to the category of secondary metabolites biosynthesis, transport, and catabolism (Q). 9.4% of the genes were assigned to more than one category (>1 cat.). 21.4% of the genes could not be assigned to a known function (S). 35% of the coding sequences in the RL1 genome were sorted in 23 main RAST subsystems and 424 subsystems (subsystem coverage). With KEGG pathway analysis genes involved in 273 pathways were identified (Supplementary Table 1). The genome was further analyzed for presence of genes known to be involved in interactions with plants using the web-based tool PIFAR and 45 genes representing 14 categories could be identified (Supplementary Table 2). Using the tool antiSMASH 17 biosynthetic gene clusters (BGC) with the potential to produce secondary metabolites, such as ectoine, erythrochelin, and heterobactin A/heterobactin S2, could be identified (Supplementary Table 3). In this study we focused on the cluster with highest similarity (>50%) to known secondary metabolite biosynthesis pathways.

**TABLE 2:** Overview of general genome properties of the isolates used in this study.

Genome properties	RL1	Djl6	BG43
Chromosome size (Mbp)	6.25	6.52	6.33
No. plasmids (size in kbp)	2 (144, 448.7)	3 (84.6, 80.9, 15.8)	3 (240.1, 266.7, 30)
GC content (%)	62.4%	62.4%	62.3%
Total genes (PGAP)	6.328	6.332	6.394
RNAs	72	77	71
NCBI Accession Numbers	NZ_CP042917, NZ_CP042916, NZ_CP042915	NZ_CP025959, NZ_CP025960, NZ_CP025961, NZ_CP025962	NZ_CP011295, NZ_CP011296, NZ_CP011297, NZ_CP011298
Reference	Kuhl et al., 2019	Xu et al., 2007; Wang et al., 2010; Táncsics et al., 2014	Rückert et al., 2015

The genome annotation of RL1 revealed several genes which have been previously identified to be involved in stress tolerance under different abiotic stress conditions, bioremediation of toxic compounds, rhizosphere colonization and (beneficial) plant-microbe interactions and were partly verified by manual annotation with blastp alignment (Supplementary Table 4). In more details, the RL1 genome harbors many genes, which can be expressed to withstand osmotic, salt, oxidative and acidic stress and are relevant for heavy metal tolerance (mercury, lead, cadmium, arsenic) and bioremediation of aromatic hydrocarbons (*alkB*, *catA*) and fossil fuels (*dszB*). Moreover, genes potentially involved in multiple drug resistance, DNA repair by phosphorothioation, antibiotic resistance and degradation of CO and hydrogen could be identified, for example the complete carbon monoxide dehydrogenase (CODH) and a [NiFe]-hydrogenase cluster. The RL1 genome annotation indicated that it is equipped with several genes which could enable it to interact with the plant and survive in the plant environment via plant hormone and siderophore production of the siderophores enterobactin, bacillibactin, arthrobactin, and heterobactin as well as nitrogen fixation, iron acquisition, phosphate solubilization, biofilm formation, and stress protection. Additionally, the RL1 genome harbors genes involved in quorum quenching, glucosinolate metabolism, aldoxime, isothiocyanate (ITC) and nitrile degradation, as well as genes important for the production of volatiles, exopolysaccharides (EPS), proteases and microbe-associated molecular patterns (MAMP). Therefore, we analyzed functional traits with focus on stress tolerance and plant–microbe interactions.

### Evaluation of Growth and Tolerance to Different Stress Factors

#### Mercury Tolerance

The RL1 genome harbors genes for alkylmercury lyase and *merR* family DNA-binding protein (Supplementary Table 4). Active growth determined by optical density was detectable in the medium with 0.001 mM mercury for djl6 and BG43. RL1 was able to grow in the medium with up to 0.01 mM mercury. RL1 and BG43 could recover from up to 1 mM mercury in the medium, whereas djl6 recovered from up to 0.1 mM mercury. The gram-positive control strain *Bacillus velezensis* FZB42 could grow in the medium with up to 0.01 mM mercury and the gram- negative control strain *Herbaspirillum frisingense* GSF30 only in medium with 0.001 mM mercury. Both control strains did not recover from medium containing 0.1 mM mercury.

#### Salt Stress Tolerance

The RL1 genome harbors genes for the complete Na+/H+ antiporter operon (Supplementary Table 4). RL1 and BG43 were able to grow in medium with 7.5% NaCl, whereas djl6 could grow up to 5.5% NaCl in the medium. Although there was no visible growth, all tested *Rhodococcus* strains were able to recover from salt stress of 15% NaCl in the medium. The gram-positive control strain *Bacillus velezensis* FZB42 could grow up to 7.5% NaCl in the medium and did not recover from medium with 15% NaCl. The gram-negative control strain *Herbaspirillum frisingense* GSF30 could grow in medium with up to 3.5% NaCl and could not recover from 7.5% NaCl in the medium.

#### pH Tolerance

Genes encoding for squalene cyclase and the ADI cluster were identified in the RL1 genome (Supplementary Table 4). The *Rhodococcus* strains were able to grow up to pH 5 and recovered after 48 h in pH 3 and 4 h in pH 2. Control strains *Herbaspirillum frisingense* GSF30 and *Bacillus velezensis* FZB42 were able to grow up to pH 5 and recovered from pH 4.

#### Osmotic Stress Tolerance

The gene cluster for ectoine biosynthesis was identified in RL1 with 75% identity to the ectoine biosynthetic cluster of *Streptomyces anulatus*. The *Rhodococcus* strains RL1, djl6, BG43, and the control strains FZB42 and GSF30 were able to grow under PEG_6000_ induced osmotic stress of –1.5 MPa, which was the tested maximum.

#### Antibiotic Resistance

Genes involved in antibiotic resistance and tellurite resistance were identified in the RL1 genome (Supplementary Table 4). RL1 was tolerant to Kanamycin up to the concentration of 96 μg/ml, Ampicillin up to 6 μg/ml, Rifampicin up to 0.025 μg/ml, but not tolerant to Vancomycin. Djl6 was tolerant to Kanamycin up to 12 μg/mL, Ampicillin up to 3 μg/mL, Rifampicin up to 0.047 μg/ml and Vancomycin up to 0.023 μg/ml. BG43 was tolerant to Kanamycin up to 48 μg/ml, Ampicillin up to 2 μg/ml, Rifampicin up to 0.023 μg/ml and Vancomycin up to 0.5 μg/ml. RL1 was able to grow on NB plates containing 100 μg/ml potassium tellurite trihydrate. The other strains were not tested for this trait.

### Traits Involved in Microbe–Plant Interactions

#### Indole-Acetic Acid Production

The genes encoding for amidase *amiE* and amine oxidase *iaaM* as well as genes involved in tryptophan metabolism were identified in the RL1 genome (Supplementary Table 4). RL1 produced 16 ± 2.6 μg/ml of IAA which is the highest amount compared to djl6 and BG43 with 10.7 ± 2.4 μg/ml and 10.9 ± 3.8 μg/ml, respectively. The positive control strain *Herbaspirillum frisingense* GSF30 produced 41 ± 9.8 μg/ml IAA.

#### Siderophore Production

**FIGURE 4:**
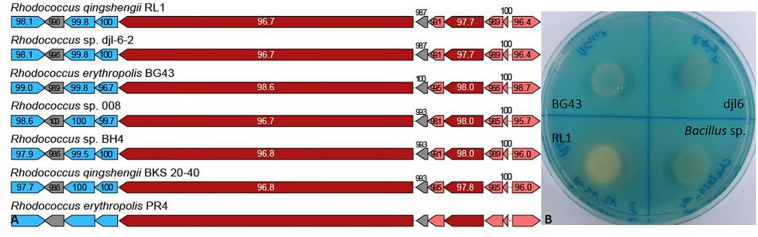
Siderophore biosynthetic gene clusters and *in vitro* assay. **(A)** Heterobactin biosynthetic gene cluster based on antiSMASH results of *Rhodococcus qingshengii* RL1 compared to the reference genome of *R. erythropolis* PR4 and other *Rhodococcus* strains. Depicted as arrows are the core biosynthetic genes in dark red, additional biosynthetic genes in light red, transport-related genes in blue and additional genes in gray. The biosynthetic genes (dark and light red) are presented in order of their appearance from right to left encoding for isochorismate synthase, isochorismatase, 2,3-dihydro-2,3-dihydroxybenzoate dehydrogenase, 2,3-dihydroxybenzoate-AMP ligase, isochorismatase, and an amino acid adenylation domain-containing protein. Numbers indicate percentage of similarity to PR4. **(B)** *In vitro* assay for siderophore production of RL1, BG43, djl6 and *Bacillus* sp. detected with Chrome Azurol Blue overlay agar. Siderophore production is indicated by color change of the medium from blue to orange.

In the RL1 genome, biosynthesis cluster for erythrochelin was identified with 57% identity and heterobactinA/heterobactin S2 identified with 100% identity compared to the heterobactin BGC of *R. erythropolis* PR4 (Figure 4A). Genes encoding for relevant proteins of the heterobactin BGC are isochorismate synthase, isochorismatase, 2,3-dihydro-2,3-dihydroxybenzoate dehydrogenase, 2,3-dihydroxybenzoate-AMP ligase, amino acid adenylation domain-containing protein and related transporter (Supplementary Table 4). RL1 produced siderophores indicated by the color change of the overlay agar from blue to orange (Figure 4B). BG43 and djl6 did not produce siderophores.

#### Phosphate Solubilization

The RL1 genome harbors genes involved in organic acid production (Supplementary Table 4). *Rhodococcus qingshengii* strains RL1 and djl6 were able to solubilize phosphate indicated by clear halo formation and SI values of 2.3 and 2.4 respectively. BG43 showed no halo formation and SI value was 2, which indicates no phosphate solubilization. Positive control *Luteibacter* sp. Cha2324a_16 showed halo formation and SI values of 2.7.

#### 1-Aminocyclopropane-1-Carboxylate Utilization

**FIGURE 5:**
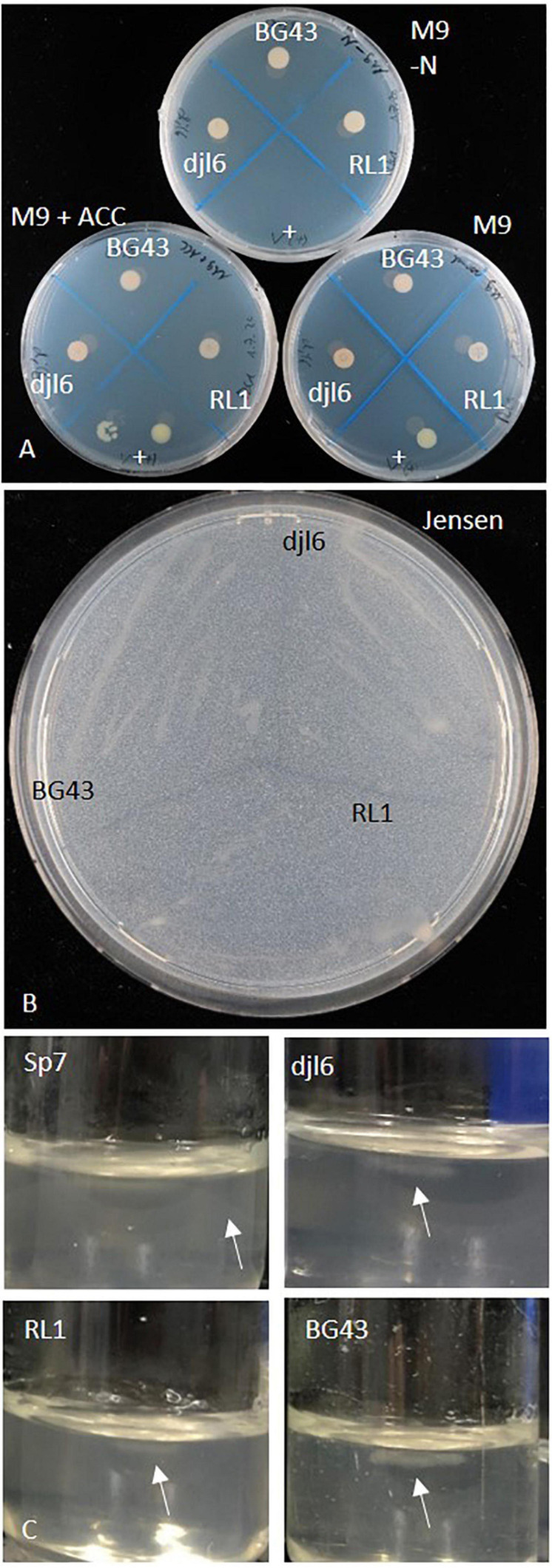
Growth characterization of *Rhodococcus* strains RL1, djl6, and BG43 on various nitrogen-free media. **(A)** M9-N, M9 (control) and M9 + ACC, **(B)** Jensen’s medium, and **(C)** Nfb-semisolid medium (Döbereiner, 1995) Sp7 = *Azospirillum brasilense* Sp7 (positive control for nitrogen fixation), + = *Variovorax* sp. (positive control for ACC deaminase activity). Arrows mark pellicle formation.

Growth on M9 and M9 with ACC and no growth on nitrogen-free M9 indicate ACC utilization. RL1, BG43, and djl6 could grow on plates with ACC, on regular M9 medium and on nitrogen-free medium. The positive control *Variovorax* sp. M92526_27 grew on M9 and M9 with ACC, but not on M9 without nitrogen (Figure 5A). ACC deaminase activity remained unclear, because *Rhodococcus* strains could also grow on M9 without nitrogen. The gene *acdS* was not present in the RL1 genome.

#### Nitrogen Fixation

The RL1 genome harbors an uncharacterized nifU-like protein (Supplementary Table 4). The strains RL1, djl6, and BG43 could grow on all tested nitrogen free media, which were nitrogen-free M9 medium (Figure 5A), Ashby’s medium, Jensen’s medium (Figure 5B) and Nfb-medium (Figure 5C). Pellicle formation in Nfb-medium was smaller compared to positive control *Azospirillum brasilense* Sp7.

#### Biofilm Formation

**FIGURE 6:**
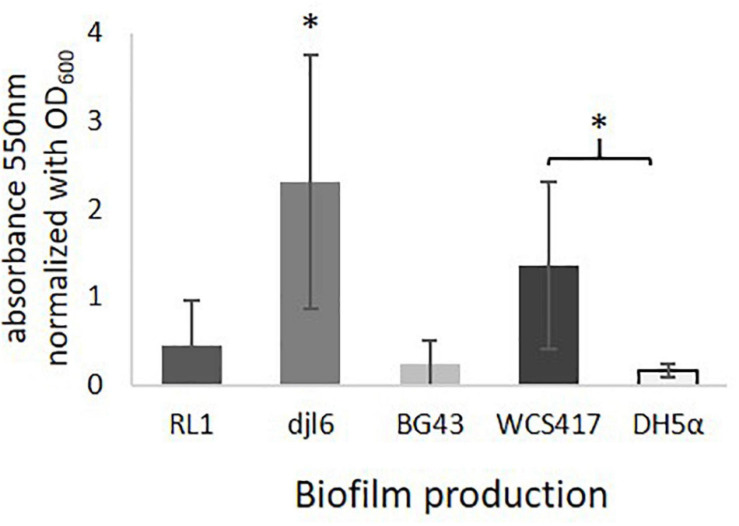
Ability to produce Biofilms with averaged results from 3 experiments normalized to OD600 = 1. Significant difference is indicated by asterisks representing ^∗^*P* < 0.05.

Genes encoding for a phosphoglucomutase and a signal peptidase I were identified in the RL1 genome (Supplementary Table 4). The strains RL1, djl6 and BG43 were able to produce biofilms in varying intensities (Figure 6), but stronger than the negative control *Escherichia coli* DH5α. Djl6 showed the strongest biofilm formation. Positive control *Pseudomonas simiae* WCS417 normalized biofilm formation was lower compared to djl6, but stronger compared to RL1 and BG43.

### Interaction With Other Microbes

#### Confrontation Assay Against Plant–Pathogenic Fungi

RL1 inhibited the plant-pathogenic fungus *Fusarium oxysporum in vitro*. The positive biocontrol strain FZB42 inhibited the plant–pathogenic fungi *Rhizoctonia solani*, *Fusarium oxysporum*, and *Fusarium culmorum* indicated by inhibition zones (Supplementary Figure 4).

#### Degradation of Synthetic and Bacterial *N*-Acyl-Homoserinelactones (AHLs)

**FIGURE 7:**
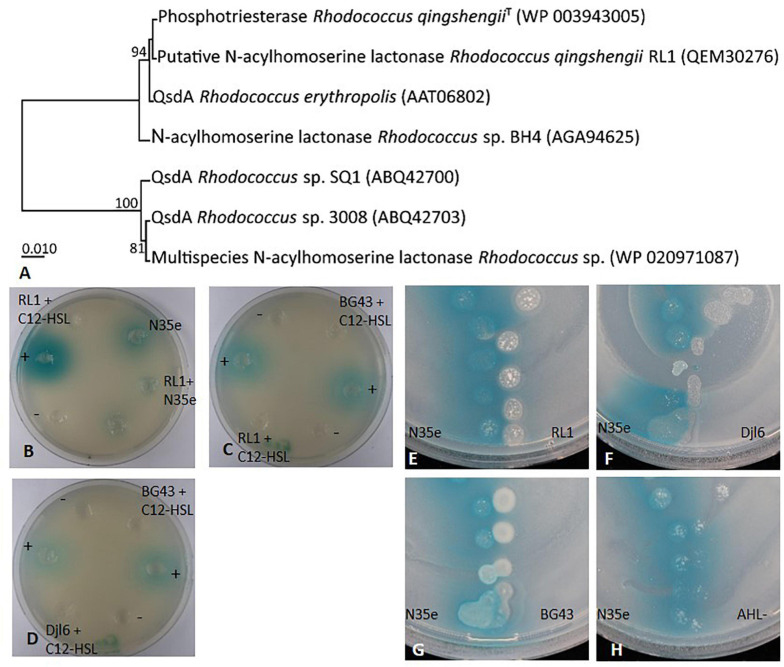
AHL degradation and quorum quenching by *Rhodococcus* strains RL1, djl6, and BG43. **(A)** UPGMA phylogenetic tree of translated *qsdA* (quorum-sensing signal degradation) gene of RL1 and related sequences from *Rhodococcus* strains. The percentage of replicate trees in which the associated taxa clustered together in the bootstrap test (1000 replicates) are shown next to the branches. The evolutionary distances were computed using the Poisson correction method. All ambiguous positions were removed for each sequence pair (pairwise deletion option). There were a total of 323 positions in the final dataset. Evolutionary analyses were conducted in MEGA X (Kumar et al., 2018). **(B–D)** Well-diffusion plate assays on NB plates all supplemented with the sensor strain A136 and X-Gal. Except for the cultures containing AHL producing strain *A. radicis* N35e, C12-HSL was added during cultivation of all bacteria. Supernatants of these cultures were added to the wells and blue color formation by the sensor strain indicated remaining AHL in the tested supernatant. NB with C12-HSL (+) served as positive control, and NB without C12-HSL (–) as negative control. **(E–H)** V-shaped assays on NB after 30 h supplemented with the sensor strain A136 and X-Gal. AHL negative mutant *A. radicis* N35e AHL- served as control. Presence of AHLs is detected by the sensor strain *Agrobacterium tumefaciens* A136 indicated by blue color change of X-Gal.

A *qsdA* gene (QEM30276) could be identified in the RL1 genome, which belongs to a class of large-spectrum quorum-quenching lactonases also present in other *Rhodococcus* sp. (Figure 7A). Therefore, AHL degradation ability was tested in RL1, djl6 and BG43 using the sensor strain A136. In this set-up it could be clearly shown that RL1, djl6 and BG43 were able to degrade synthetic C12-HSL (Figures 7B–D). Additionally, co-culturing of *Acidovorax radicis* N35e with RL1 resulted in no visible blue color formation by the sensor strain, indicating degradation of produced AHL (Figure 7B). Finally, V-shaped spotting of *Acidovorax radicis* N35e and RL1, djl6 and BG43 showed an inhibition of blue color formation where strains were in direct contact (Figures 7E–H).

### Rhizosphere Competence

#### Root Colonization in Axenic System

**FIGURE 8:**
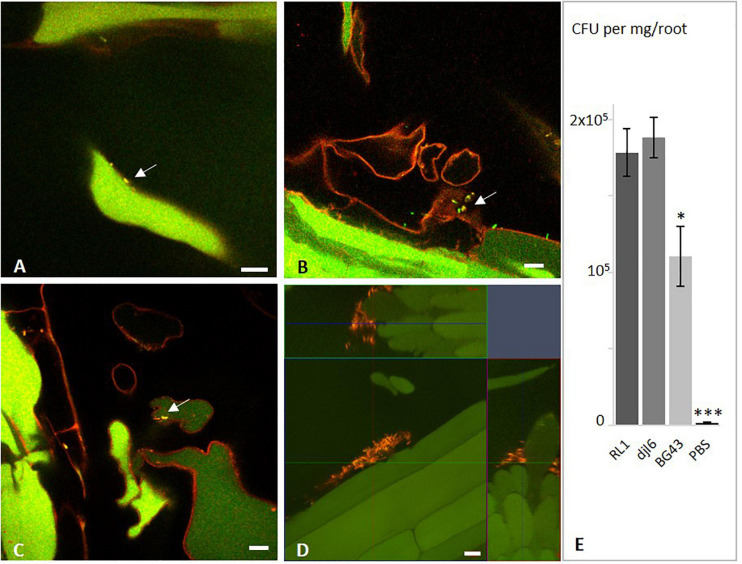
Interaction of *Rhodococcus* strains RL1, BG43, and djl6 with *Eruca sativa* roots. *In situ* detection of root colonization of *Eruca sativa* by *Rhodococcus* strains visualized by fluorescence *in situ* hybridization (FISH). **(A)** RL1, **(B)** BG43, and **(C)** djl6 on 1 week old *Eruca sativa* roots grown in axenic quartz-sand system. **(D)** RL1 on 2 weeks old *Eruca sativa* roots grown on MS agar without additional sucrose. **(E)** Quantification of root colonization 1 week after inoculation. Arrows indicate bacterial cells identified by yellow color from overlaying channels of probes EUB (green) and HGC (red). Root autofluorescence is assigned in green and red. Scale bar represents 10 μm. Significant difference is indicated by asterisks representing ^∗^*P* < 0.05 and ^∗∗∗^*P* < 0.001.

Root colonization was analyzed with fluorescence *in situ* hybridization using probes EUB Mix Fluos and HGC69A Atto550 or HGC69A Cy3. Single cells of RL1 could be found on the root surface of its host plant Rucola (*Eruca sativa* L.) when grown in the axenic system (Figures 8A–C), while dense cell patches were identified on roots from MS agar plates (Figure 8D). Similar colonization patterns were found for strains djl6 and BG43. All strains were localized rather in the basal mature part of the root in areas of emergence of root hairs. No endophytic colonization was observed.

Quantitative estimation based on CFU/mg root mass (Figure 8E) showed significantly higher colonization numbers for RL1 and djl6 (*p*-value = 0.012) than BG43. Djl6 showed a trend toward higher root colonization compared to RL1. All strains were significantly higher than the uninoculated control.

## Discussion

The genus *Rhodococcus* is frequently found in the plant microbiome (Francis and Vereecke, 2019; Vereecke et al., 2020). Therefore, it is important to analyze and understand functions of the plant-associated members of this genus, such as RL1 isolated from *Eruca sativa* leaves. Additionally, as mentioned in the introduction, the genus *Rhodococcus* is well-known for stress tolerant strains (Pátek et al., 2021). For these reasons, we wanted to elucidate genomic properties with a special focus on functional analyses of stress tolerance and interaction with plants to understand the possible functions that the plant-associated *R. qingshengii* RL1 could provide within the plant holobiont and also compare it to the closely related strains djl6 and BG43.

### RL1 Genome Harbors Several Genes Involved in Survival and Tolerance to Different Stress Conditions

Genes involved in acidic pH tolerance were identified in RL1, either involved in the production of the compound squalene, such as squalene cyclase, a precursor of hopanoid (Schmerk et al., 2011) or based on the expression of the ADI cluster (in the presence of arginine). The latter is a mechanism to overcome acidic stress often found in gram-positive bacteria (Cotter and Hill, 2003). The experimental evidence proved the ability of *Rhodococcus* strain RL1 to survive and recover from acidic pH conditions. This trait was also shared by the closely related strains djl6 and BG43, indicating that this trait may be widespread amongst the genus *Rhodococcus*. In the genus *Rhodococcus* tolerance to acidic pH was reported for *R. qingshengii* BBG1 (Benedek et al., 2012) and for the mammalian pathogen *Rhodococcus equi*, which can withstand a pH of 4 (Benoit et al., 2000). Conventional agricultural practices and soil exploitation can lead to increased soil acidity (Goswami et al., 2017). Therefore, acidic pH tolerance is an important trait of plant-associated and soil bacteria to maintain a functional plant microbiome also under acidic soil conditions.

Ectoine is a compound associated with osmoregulation in bacteria (Bremer and Krämer, 2019) and important for survival during osmotic stress. The gene cluster for ectoine biosynthesis and transporters were identified in RL1, indicating the ability of RL1 to synthesize ectoine under osmotic stress. Alternative to biosynthesis, bacteria can take up compatible solutes, such as proline or betaine from their environment (Bremer and Krämer, 2019). Genes encoding the respective transporters were found in the RL1 genome. Additionally, the full operon of Na+/H+ antiporter was identified in the RL1 genome, which could play a role in salt stress tolerance (Liu et al., 2016; Bhat et al., 2020). Results of the *in vitro* experiments of the tested *Rhodococcus* strains growing under high salt and osmotic stress confirmed previous reports of osmotic and salt stress tolerant members of the genus *Rhodococcus*. For example, an upregulation of genes involved in ectoine biosynthesis was observed in *Rhodococcus jostii* RHA1 under desiccation (LeBlanc et al., 2008) and rapid adaptation to salt stress was described for *R. erythropolis* DSM 1069 (De Carvalho et al., 2014). Moreover, plant associated bacteria tolerant to osmotic and salt stress could also be beneficial for the plant via support of ion homeostasis (Bhat et al., 2020; Salas-González et al., 2021) and upregulation of osmoprotective compound biosynthesis in the plant. For example, *Bacillus* sp. can directly influence proline biosynthesis in plants to improve osmotolerance (Kaushal and Wani, 2016; Bhat et al., 2020).

Heavy metals, such as mercury, are highly persistent environmental pollutants and a threat to all living organisms (Boyd and Barkay, 2012). Organomercury compounds were used in several agricultural applications, for example as common pest control agent in the 1900’s. Although its use has been banned in several countries, it is still used in Australia to treat the plant pathogenic fungus *Ceratocystis paradoxa* (Schneider, 2021). Mercury resistant bacteria can convert organomercury compounds or Hg(II) to gaseous Hg(0) to reduce the mercury concentration in their environment (Boyd and Barkay, 2012). Mercury resistance was described in *R. erythropolis* BD2 and *Pseudomonas fluorescens* SBW25 to be located and transferred on a plasmid (Dabrock et al., 1994; Hall et al., 2020) containing the *mer*-operon (Boyd and Barkay, 2012). Loss of this plasmid caused a loss of mercury resistance (Dabrock et al., 1994; Hall et al., 2020). However, in RL1 the identified mercury resistance genes, such as a transcriptional regulator *MerR* and a unique alkylmercury lyase involved in the degradation of toxic organomercury compounds (e.g., MeHg) (Schaefer et al., 2004), are located in the chromosome. We report for the first time that mercury tolerance is also present in an isolate of *R. qingshengii* based on the results of the *in vitro* experiment. BG43 and RL1 were both able to survive up to 1 mM of mercury in the growth medium. Survival and detoxification of heavy metals have been reported for other members of the genus *Rhodococcus* (Trivedi et al., 2007; Irawati et al., 2012), emphasizing the exceptional stress tolerance of this genus. Heavy metal resistance in bacteria in combination with a close association with plants could indicate the adaptation to toxic heavy metal residues of such compounds previously used as pesticides.

Apart from tolerance to heavy metals, we could identify several operons in the genome of RL1 which show that this bacterium has the ability to survive under selective environmental conditions by metabolizing trace gasses like CO and H_2_. Comparison of deduced amino acid sequences revealed that the identified CODH belongs to the functional type1-CODH enzymes, which catalyze the unidirectional conversion of CO to CO_2_ (King and Weber, 2007). This type of enzyme has been extensively studied in aerobic CO-oxidizers, or carboxydotrophic Actinobacteria (Quiza et al., 2014). Sequence similarity revealed that the identified [NiFe]-hydrogenase cluster belongs to the high-affinity group 1 h/5 Actinobacteria type of hydrogenases which have been shown to scavenge electrons from tropospheric H_2_ to sustain aerobic respiration during starvation (Constant et al., 2011; Greening et al., 2016). Interestingly, less is known about plant associated atmospheric H_2_-oxidizing bacteria. Atmospheric H_2_ may serve as the maintenance energy during starvation and sporulation of high-affinity H_2_-oxidizing Actinobacteria, providing them the advantage of survival in plant tissues, as was shown for endophytic *Streptomyces* spp. (Greening et al., 2016; Kanno et al., 2016). The simultaneous presence of the carbon monoxide dehydrogenase (CODH) genes and the [NiFe]-hydrogenase cluster indicate that RL1 can use CO and H_2_ as energy source.

The functional annotation of RL1 genome also revealed that it harbors genes involved in multidrug resistance, tellurite resistance and antibiotic resistance. Tellurite is a metalloid often used as antibiotic compound in *in vitro* experiments and is toxic to eukaryotic and prokaryotic cells (Chien and Han, 2009). Resistance against tellurite can be mediated by a reduction of tellurite (TeO_3_^2–^) to elemental tellurium, indicated by the color change of the colonies, which was also observed in RL1. Moreover, the RL1 genome harbors genes potentially involved in protection against oxidative stress. These genes could be involved in detoxification of tellurite, because the toxicity of tellurite is eventually caused through intracellular generation of reactive oxygen species (ROS) (Pérez et al., 2007).

*In vitro* tests with antibiotics revealed resistance of RL1 against kanamycin and ampicillin, whereas djl6 and BG43 are more resistant to rifampicin and vancomycin respectively. Antibiotic resistance was mainly investigated and is widespread in the horse pathogen *Rhodococcus equi* (Giguère et al., 2017), because of its relevance in livestock animal infections. Antibiotic resistance in plant-associated *Rhodococcus* species was not intensively studied yet and could confer them a competitive advantage in surviving against other antibiotic-producing microbes in specialized niches like the rhizosphere (Raaijmakers et al., 2009; Mendes et al., 2013).

### RL1 Genome Reveals Successful Interaction and Survival Strategies in Association With Plants

The genome annotation of RL1 and functional analysis revealed a large repertoire of traits involved in plant–microbe and microbe–microbe interactions, which can be relevant for the role of RL1 in the plant microbiome.

An important trait of plant-associated bacteria is the ability to colonize plant roots to facilitate, e.g., the exchange of metabolites (Kloepper and Beauchamp, 1992; Pandit et al., 2020). For successful root colonization it can be beneficial for the bacteria to be able to produce biofilms (Pandit et al., 2020), which was demonstrated for RL1, djl6, and BG43. Accordingly, the RL1 genome harbors genes encoding for enzymes involved in biofilm formation. Qualitative evaluation of rhizosphere competence revealed that all three strains were able to colonize the roots of *E. sativa* epiphytically. However, quantitative evaluation revealed that RL1 and djl6 had significantly more CFUs per mg *E. sativa* root than BG43, which indicates a better root colonization ability of *R. qingshengii* species. Verification of endo- or epiphytic leaf colonization of RL1 analyzed with FISH (data not shown) did not deliver clear results due to high auto-fluorescence of the leaves and transformation of fluorescent markers in RL1 was not successful. Therefore, final conclusions upon leaf colonization of RL1 cannot be drawn.

The leaves of *Brassicacea*, such as Rucola (*Eruca sativa* L.) contain glucosinolates (GSLs), which are sulfur-containing secondary metabolites involved in the protection of plants against herbivores (Textor and Gershenzon, 2009; Bell et al., 2015). Since RL1 was isolated from the leaves of Rucola (*Eruca sativa* L.), we were interested if the genome reveals some interesting information about its ability to metabolize glucosinolates. Our results showed that the genome harbors genes potentially involved in the metabolic pathways of GSLs, such as myrosinase, methionine sulfoxide reductase (*msrA*, *msrB*) or aldoxime dehydratase *oxd* (Supplementary Table 4). Degradation of GSLs was investigated for gut microbes regarding beneficial effects of ITC production as a chemoprotective function against cancer (Mullaney et al., 2013a,b; Bessler and Djaldetti, 2018; Mokhtari et al., 2018). An *in vitro* experiment with Rucola (*Eruca sativa* L.) leaf extract and pure GSLs (data not shown) did not reveal clear and consistent results on GSL synthesis, bioconversion or degradation by RL1, djl6, and BG43.

The RL1 genome harbors genes related to the production of volatiles, exopolysaccharides and proteases, which are important in microbial communication, plant colonization and microbial detection by the host (Flemming et al., 2016; Netzker et al., 2020). The chemotaxis protein CheY relevant for the transmission of sensory signals from the chemoreceptors to the flagella motors, which is additionally a microbe-associated molecular pattern (MAMP) (Paul et al., 2010) was identified in the RL1 genome. As *Rhodococcus* is a non-motile genus CheY has a rather different function, e.g., in sensory signal transduction in another pathway or interaction with the plant. Additionally, in the RL1 genome genes encoding for a LacI transcription regulator and an aldo-keto reductase were identified, which were found to be enriched in genomes of plant beneficial microbes (Levy et al., 2018).

Plant associated bacteria in general can influence root growth, germination, flowering and developmental stages via balancing or producing plant hormones, such as gibberellin (Kang et al., 2014; Panke-Buisse et al., 2017; Salazar-Cerezo et al., 2018) or indole-3-acetic acid (IAA) (Finkel et al., 2020). In the *in vitro* assay RL1 produced a higher amount of IAA in comparison to the strains djl6 and BG43. The best-known pathway for IAA production includes the enzyme indolepyruvate decarboxylase (*ipdC*), which is not present in the RL1 genome. Instead genes of the alternative indole-3-acetamide pathway for IAA production (Spaepen et al., 2007) were identified in the RL1 genome. The ability to produce IAA *in vitro* was not only shown for RL1 but also in another *R. qingshengii* strain (Hasuty et al., 2018) and other members of the genus *Rhodococcus* (Francis and Vereecke, 2019). Bacterial production of IAA can be beneficial for the plant by increasing the root system (Spaepen and Vanderleyden, 2011) and balancing IAA production is an important function of the root microbiome (Finkel et al., 2020). Gibberellin production is encoded by a conserved operon, which was characterized in α- and β-proteobacteria (Nagel et al., 2018). Essential parts of the gibberellin operon were identified in the RL1 genome. To our knowledge, this is the first report about the presence of genes of the gibberellin operon in any Actinobacteria. Some plant-pathogenic bacteria produce bioactive GA4, which can have a detrimental effect on seedling development. Beneficial bacteria only produce the precursor GA9 as they lack the cytochrome P450 (CYP115) for the final step in the production of the bioactive GA4 (Nagel and Peters, 2017). As RL1 also lacks the cytochrome P450 (CYP115) this indicates its allocation to the plant beneficial bacteria. Verification of the production of gibberellin by RL1 with gas chromatography was beyond the scope of this work.

The bacterially produced polyamine spermidine increases biofilm formation and overall bacterial fitness (Xie et al., 2014; Liu et al., 2016). Additionally, it is the plant growth-promoting compound in strains such as *B. subtilis* OKB105 or *Klebsiella* sp. D5A (Xie et al., 2014; Liu et al., 2016) and the upregulation of spermidine export proteins in *Stenotrophomonas rhizophila* DSM14405 upon salt stress in combination with exposure to root exudates emphasizes the role of spermidine as key substance in stress protection in roots (Alavi et al., 2013). Presence of genes encoding for the enzymes involved in the biosynthesis of spermidine, such as arginine decarboxylase, agmatinase, spermidine synthase in the RL1 genome indicate the ability of RL1 to function as stress-protecting agent and support plants under abiotic stress.

The degradation of 1-amino-cyclopropane-1-carboxylate (ACC), the precursor of the plant hormone ethylene, by bacterial ACC deaminase can protect the plant from detrimental effects of long exposure to ethylene (Glick, 2012; Dubois et al., 2018). In a standard *in vitro* assay RL1, djl6, and BG43 were able to grow on nitrogen-free M9 plates with ACC in the medium, indicating ACC deaminase activity (Figure 5A). However, the essential gene *acdS* encoding for ACC deaminase is missing in the RL1 genome. Additionally, all three tested *Rhodococcus* strains were able to grow on all tested nitrogen free media. The results indicate that the isolates grow on N-free media through utilization of atmospheric nitrogen rather than using ACC as a nitrogen source. Biological nitrogen fixation is defined as the bacterial conversion of dinitrogen to ammonia through the expression of canonical *nif* gene products (Dos Santos et al., 2012; Higdon et al., 2020). In the RL1 genome the SUF system FeS assembly protein of the *nifU* family was identified (MSMEG_2718, Supplementary Table 4), which stabilizes the nitrogenase complex and is relevant for diazotrophy especially under low temperature conditions (Suyal et al., 2014). The *nifH* gene was previously identified in a diazotrophic *R. qingshengii* strain (Suyal et al., 2014; Joshi et al., 2019) and used as molecular marker to directly link to a diazotrophic lifestyle. However, no *nifH* gene was identified in the RL1 genome. In a large scale genome analysis Higdon et al. (2020) identified three distinct groups of diazotrophic bacteria defined by *nif* gene content and structural variation. The genus *Rhodococcus* was classified as DS-negative (=no Dos Santos model *nif* gene homolog present in genome). This implies the presence of alternative *nif* genes and metabolic pathways relevant for nitrogen fixation in *Rhodococcus* genomes beyond the currently known models. Transcriptome analysis and mutant construction would reveal insights to alternative nitrogen fixation mechanisms in RL1 as representative of the genus *Rhodococcus*.

Siderophores not only chelate iron and have beneficial effects in plant growth, they are also involved in bioremediation, function as biosensors and are relevant in microbial competition and defense against other microbes, which can lead to a beneficial biocontrol effect for the plant (Ahmed and Holmström, 2014; Gu et al., 2020; Pollak and Cordero, 2020). Genes involved in iron acquisition and siderophore production were identified in the RL1 genome, for example for the siderophore heterobactin, which is unique to the *Rhodococcus* genus (Carrano et al., 2001; Bosello et al., 2013; Khilyas et al., 2020). The *in vitro* assay for siderophore production was positive for RL1, corroborating that the identified genes were actually expressed. These results were in contrast to djl6 and BG43, where the functional analysis was negative. Iron acquisition and ferrous iron transport can occur via two systems, the FeoABC and EfeUOB transporters (Lau et al., 2016). The EfeUOB was reported to be low-pH-induced (Cao et al., 2007) and was predicted in the genome of a *Leptospirillum* sp. tolerant to acidic pH (Osorio et al., 2008). RL1 harbors the genes encoding for the EfeUOB operon, which could contribute to the low pH tolerance of RL1, because it allows iron acquisition also under low pH.

The RL1 genome harbors genes relevant for organic acid production, which are involved in phosphate solubilization and genes potentially relevant for phosphate metabolism and transport. However, genes involved in gluconic acid production, which is the main driver in phosphate solubilization could not be identified (Rodríguez et al., 2006). Despite of that, the *in vitro* assay for this trait was positive for RL1 which suggests the presence of alternative organic acids involved in phosphate solubilization. Phosphate solubilization capacity was previously reported for *Rhodococcus globerulus* isolated from *Plectranthus amboinicus* (Murugappan et al., 2017).

### RL1 Genome Reveals Competitive Potential Against Other Microorganisms

Members of the genus *Rhodococcus* have been reported to show antifungal activity *in vitro* against plant-pathogenic fungi (Chiba et al., 1999; Iwatsuki et al., 2007; Santos et al., 2020) and RL1 reduced growth of *F. oxysporum in vitro*, but showed no inhibition against *R. solani* and *F. culmorum* (Supplementary Figure 4). Further studies using model plants will reveal the full potential of RL1 as biocontrol agent against plant pathogenic fungi.

Degradation or interference with quorum sensing molecules can disturb bacterial communication and is called quorum quenching (Dong et al., 2001). The *qsdA* gene, encoding for a *N*-acyl-HSL lactonase was first described by Uroz et al. (2003) for the strain *R. erythropolis* W2 and could also be identified in the RL1 genome (Figure 7A). Moreover, the RL1 genome harbors a two-component transcriptional AHL responsive regulator from the LuxR family. However, as RL1 is not producing AHLs this regulator is likely a so-called LuxR-solo, which allows bacteria to respond to quorum sensing signals from neighboring cells without itself contributing to signal synthesis. This was also previously described for the genus *Rhodococcus* and other gram-positive bacteria (Subramoni and Venturi, 2009; Santos et al., 2012). *In vitro* experiments showed the ability of RL1 to degrade AHLs. To our knowledge, this is the first report of an AHL-degrading *R. qingshengii*. Also it is the first description of functional AHL degradation by BG43, which was previously reported only to interfere with the quinolone signal of *Pseudomonas aeruginosa* (Müller et al., 2014). Quorum quenching ability was intensively studied in *R. erythropolis* R138 (Cirou et al., 2007; Barbey et al., 2013; Latour et al., 2013; Kwasiborski et al., 2015), which was able to reduce the soft-rot pathogen *Pectobacterium* in potatoes and most likely use the degraded AHLs as carbon source. Quorum quenching can also be a beneficial trait in other crop-pathogen systems as reported for example in *Pseudomonas segetis* (Rodríguez et al., 2020) or *Bacillus thuringensis* (Dong et al., 2004). Further analysis of RL1 quorum quenching abilities, e.g., against plant pathogens such as *Pectobacterium* or *Pseudomonas syringae*, would reveal its full potential as plant biocontrol agent.

### Genome Comparison of Related *R. erythropolis* and *R. qingshengii* Isolates Show Potential for Re-classification of Clade Members

*Rhodococcus* is a genus well-known for its high potential to produce versatile secondary metabolites and the RL1 genome annotation confirms previous studies (Ceniceros et al., 2017; Thompson et al., 2020). The number of genes from the genome of RL1, which were assigned to the COG group for secondary metabolites, were higher compared to other bacteria, for example *Stenotrophomonas* or *Enterobacter* (Alavi et al., 2014; Andrés-Barrao et al., 2017). Additionally, 17 BGC for secondary metabolites were identified in RL1. The average number of BGCs in the *R. erythropolis* clade are 13–24 BGCs and are mostly shared by *R. erythropolis* and *R. qingshengii* strains (Thompson et al., 2020). Four BGC cluster were highly conserved among the *R. erythropolis* clade and three of them were also identified in RL1 (Supplementary Table 3). The remaining unknown BGCs in the RL1 genome are potentially capable of producing novel compounds which could be analyzed in future studies.

*Rhodococcus* is a heterogeneous genus with eight identified phylogenetic clades (Alvarez, 2019). Phylogenetic analysis based on complete genome sequences of the *R. erythropolis* clade reveals a clear separation into two subgroups at an ANI value of 97% (Figure 1 and Supplementary Figure 2). The first one includes sequences belonging to only *R. erythopolis*, the second includes *R. qingshengii* and *R. erythropolis* strains. Based on the clear separation we can confirm previous recommendations to separate the *R. erythropolis* clade into the two groups consisting of the species *R. qingshengii* and *R. erythropolis* respectively (Sangal et al., 2016; Khilyas et al., 2020; Thompson et al., 2020). We also suggest that the *R. erythropolis* strains assigned to the *R. qingshengii* group should be re-named as previously recommended (Sangal et al., 2016; Thompson et al., 2020). The genomes of RL1 and djl6 were clearly identified as belonging to the *R. qingshengii* cluster (Xu et al., 2007; Kuhl et al., 2019) and had more genes in common with each other than with BG43 (Figure 2), whereas the BG43 genome was classified as *R. erythropolis* (Müller et al., 2014; Rückert et al., 2015). Despite the clear separation the strains RL1 and BG43 had many functional traits in common, indicating a close functional overlap between the species. At the same time, djl6 and RL1 showed different results in the *in vitro* experiment for siderophore production and mercury tolerance, indicating differences on the genetic and functional level also within the species *R. qingshengii* (Figure 4). RL1 showed overall the best performance in the tested traits. This emphasizes the importance of RL1 and the necessity to analyze the genetic and functional potential of individual strains to understand the role also of lesser known members of the plant microbiome.

## Conclusion and Outlook

The study shows the remarkable genomic potential of the isolate *R. qingshengii* RL1 for tolerating various abiotic stresses, plant–microbe and microbe–microbe interactions, many of which could be confirmed by functional analysis *in vitro*. By this thorough characterization we aim to contribute to a better understanding of relevant attributes for interactions in the plant holobiont as well as provide selection criteria for using strains, such as RL1, in specific agricultural or biotechnological applications. Furthermore, we provided phylogenetic evidence based on whole genome comparisons to justify a taxonomic separation of the *R. erythropolis* and *R. qingshengii* cluster and re-name some members of the *R. erythropolis* cluster. However, the functional analysis also indicates many shared traits between the two species *R. qingshengii* and *R. erythropolis*, some of them also described for the first time for the strains djl6 and BG43, but also different traits within the same species. Further experiments involving inoculation of different plants with RL1 under various conditions, which were beyond the scope of this study, would reveal insights into its plant beneficial functions. This could be coupled with transcriptome analysis of RL1 to reveal such intriguing aspects as an alternative nitrogen fixation pathway. Further investigation of the quorum quenching ability against various plant–pathogenic bacteria could advance the understanding of the role of RL1 in biological control.

## Data Availability Statement

The datasets presented in this study can be found in the NCBI database. Accession numbers are for *Rhodococcus qingshengii* RL1 NZ_CP042917, NZ_CP042916, NZ_CP042915, for *Rhodococcus qingshengii* djl6 NZ_CP025959, NZ_CP025960, NZ_CP025961, NZ_CP025962, and for *Rhodococcus erythropolis* BG43 NZ_CP011295, NZ_CP011296, NZ_CP011297, andNZ_CP011298.

## Author Contributions

TK, SC, and MR contributed to conception and design of the study. TK performed the experiments and wrote the first draft of the manuscript. JU contributed parts of the experimental data. SC and TK analyzed the data. SC and MR wrote sections of the manuscript. All authors contributed to manuscript revision, read, and approved the submitted version.

## Conflict of Interest

The authors declare that the research was conducted in the absence of any commercial or financial relationships that could be construed as a potential conflict of interest.

## Publisher’s Note

All claims expressed in this article are solely those of the authors and do not necessarily represent those of their affiliated organizations, or those of the publisher, the editors and the reviewers. Any product that may be evaluated in this article, or claim that may be made by its manufacturer, is not guaranteed or endorsed by the publisher.
