# Supplementary material for: Genome-Based Characterization of Plant-Associated Rhodococcus qingshengii RL1 Reveals Stress Tolerance and Plant–Microbe Interaction Traits
Source: Front Microbiol. 2021 Aug 18;12:708605. doi: 10.3389/fmicb.2021.708605 (PMC8416521; doi:10.3389/fmicb.2021.708605)
Supplement: Supplementary file 2 [file Data_Sheet_1.docx]

Supplementary Material

# Supplementary Table

**Table S1:** List of annotated KEGG pathways in *Rhodococcus qingshengii* RL1.

**(Separate excel list)**

**Table S2:** List of annotated genes in *Rhodococcus qingshengii* RL1 with the Software PIFAR.

**(Separate excel list)**

**Table S3:** List of annotated genes in *Rhodococcus qingshengii* RL1 with the Software antiSMASH.

|  | **Type** | **From (bp)** | **To (bp)** | **Most similar known cluster** | **Class of secondary metabolites** | **Similarity** |
| --- | --- | --- | --- | --- | --- | --- |
| 1 | ectoine | 965,135 | 975,533 | ectoine | Other | 75% |
| 2 | terpene | 1,174,497 | 1,194,805 | isorenieratene | Terpene | 25% |
| 3 | NRPS | 1,274,976 | 1,329,636 | monensin | Polyketide | 5% |
| 4 | NRPS | 1,332,756 | 1,415,066 | rifamorpholine A / rifamorpholine B / rifamorpholine C / rifamorpholine D / rifamorpholine E | Polyketide | 4% |
| 5 | NRPS | 1,634,393 | 1,697,371 | coelichelin | NRP | 27% |
| 6 | NRPS,terpene | 1,730,598 | 1,783,490 | SF2575 | Polyketide:Type II + Saccharide:Hybrid/tailoring | 6% |
| 7 | NRPS | 2,071,856 | 2,126,033 | heterobactin A / heterobactin S2 | NRP | 100% |
| 8 | NRPS-like | 2,390,492 | 2,432,746 | thiolutin | NRP | 8% |
| 9 | NRPS | 2,824,057 | 2,931,597 | chloramphenicol | NRP | 17% |
| 10 | NRPS | 3,572,754 | 3,618,512 | hygromycin A | Saccharide | 9% |
| 11 | NRPS | 3,815,523 | 3,872,232 | erythrochelin | NRP | 57% |
| 12 | NRPS-like | 4,205,438 | 4,249,331 |  |  |  |
| 13 | T1PKS | 4,378,613 | 4,424,648 | kirromycin | NRP + Polyketide:Modular type I + Polyketide:Trans-AT type I | 8% |
| 14 | T1PKS | 4,557,040 | 4,601,965 |  |  |  |
| 15 | LAP | 4,692,380 | 4,722,476 | diisonitrile antibiotic SF2768 | NRP | 11% |
| 16 | bacteriocin | 4,882,807 | 4,894,737 | branched-chain fatty acids | Other | 75% |
| 17 | lanthipeptide | 5,270,044 | 5,292,626 |  |  |  |
| 18 | butyrolactone | 5,522,805 | 5,533,692 |  |  |  |

**Table S4:** List of annotated genes in *Rhodococcus qingshengii* RL1. Functional annotation of the genome was performed with RAST, PIFAR and antiSMASH softwares.

| **Category** | **Annotated Genes and Cluster** | **RL1 gene ID** |
| --- | --- | --- |
| **Osmotic stress** |  |  |
|  | hydroxyacid dehydrogenase | D6M20_07215 |
|  | L-ectoine synthase | D6M20_07220 |
|  | Diaminobutyrate--2-oxoglutarate transaminase (EC 2.6.1.76) | D6M20_07225 |
|  | glycerol uptake facilitator protein (aquaporine family) | D6M20_03015 |
|  | Glycine betaine transporter OpuD | D6M20_04180 |
|  | L-Proline/Glycine betaine transporter ProP | D6M20_07250, D6M20_20860 |
|  | High-affinity choline uptake protein BetT | D6M20_22060 |
| **Salt stress** | Na+/H+ antiporter A and Na+/H+ antiporter B | D6M20_17510 |
|  | Na+/H+ antiporter C | D6M20_17515 |
|  | Na+/H+ antiporter D | D6M20_17520 |
|  | Na+/H+ antiporter E | D6M20_17525 |
|  | Na+/H+ antiporter F | D6M20_17530 |
|  | Na+/H+ antiporter G | D6M20_17535 |
| **Oxidative stress** | Catalase KatE-intracellular protease (EC 1.11.1.6) | D6M20_26210 |
|  | Catalase-peroxidase KatG (EC 1.11.1.21) | D6M20_19015 |
|  | Catalase KatE (EC 1.11.1.6) | D6M20_22570 |
|  | superoxide dismutase [Mn] | D6M20_23995 |
|  | sigmafactor SigB , SigF | D6M20_00855 (plasmid), D6M20_01170 (plasmid), D6M20_11750, D6M20_17130, D6M20_18680 |
|  | bacterial hemoglobin gene | D6M20_07015 |
| **pH tolerance** | squalene cyclase | D6M20_23450 |
|  | Arginine deiminase (arcA) | D6M20_04155 |
|  | ornithine carbamoyltransferase (arcB) | D6M20_09510 |
|  | ornithine/arginine antiporter (arcD) | D6M20_24210 |
|  | arginine pathway regulatory protein of the ArgR-AhrC family (argR) | D6M20_09515 |
| **DNA phosphorothioation** | Cysteine desulfurase *DndA* | D6M20_05845 |
|  | DNA sulfur modification protein *DndB* | D6M20_05850 |
|  | DNA phosphorothioation system sulfutransferase *DndC* | D6M20_05855 |
|  | DNA sulphur modification protein *DndD* | D6M20_05860 |
|  | *DndE* | D6M20_05865 |
|  | DNA phosphorothioation-associated putative methyltransferase | D6M20_05870 |
|  | DNA phosphorothioation-associated protein 4 | D6M20_05875 |
| **Multidrug resistance** | Multi_Drug_Res (PF00893) family | D6M20_06295 |
|  | DAED/DEAH box helicase family protein | D6M20_05885 |
|  | GIY-YIG nuclease family protein | D6M20_05895 |
|  | plasmid pRiA4b ORF-3 family protein | D6M20_05905 |
| **Antibiotic resistance** | vancomycin resistance protein | D6M20_19620 |
|  | beta-lactamase | D6M20_27815 |
|  | Metal-dependent hydrolases of the beta-lactamase superfamily III | D6M20_06650 |
|  | Beta-lactamase class C-like and penicillin binding proteins (PBPs) superfamily | D6M20_07770, D6M20_12855, D6M20_15185, D6M20_15195, D6M20_20105, D6M20_21115 |
|  | rifampicin monooxygenase | D6M20_14905 |
| **Tellurite resistance** | Tellurium ion resistance protein TerA | D6M20_04715 |
|  | Tellurite resistance TerB | D6M20_21770 |
|  | tellurium ion resistance protein TerD | D6M20_21785, **D6M20_21815**, D6M20_30505, **D6M20_05745** |
| **Heavy metal resistance/detoxification** | copper-translocating protein | D6M20_04250 |
|  | Cytoplasmic copper homeostasis protein CutC | D6M20_06255 |
|  | ATPase:Heavy metal (Lead, cadmium, zinc, mercury) translocating P-type ATPase | D6M20_04240, D6M20_00235 |
|  | heavy-metal-associated domain-containing protein/heavy metal transport/detoxification protein | D6M20_04250 |
|  | Copper resistance protein CopD | D6M20_06885 |
|  | Arsenical pump-driving ATPase (EC 3.6.3.16) TEMP | D6M20_00060 |
|  | Arsenical resistance operon trans-acting repressor ArsD | D6M20_00065 |
|  | lead, cadmium, zinc, arsenical resistance proteins | D6M20_19920, D6M20_00050, |
|  | IS110 family transposase | D6M20_28370 |
|  | alkylmercury lyase | D6M20_28375 |
|  | *merR* family DNA-binding protein | D6M20_28380 |
| **CO degradation** | CoxL, large subunit, Xanthine dehydrogenase, molybdenum binding subunit (EC 1.17.1.4) | D6M20_09010 |
|  | CoxM, middle subunit, Xanthine dehydrogenase, FAD binding subunit (EC 1.17.1.4) | D6M20_09015 |
|  | CoxS, small subunit, carbon monoxide dehydrogenase | D6M20_09005 |
|  | Carbon monoxide oxidation accessory protein CoxD | D6M20_07265, D6M20_16125 |
|  | Carbon monoxide oxidation accessory protein CoxE | D6M20_07270, D6M20_16120 |
|  | Xanthine and CO dehydrogenases maturation factor, XdhC/CoxF family | D6M20_25450 |
| **Hydrogen utilization** | [NiFe] hydrogenase metallocenter assembly protein HypE | D6M20_12420 |
|  | [NiFe] hydrogenase metallocenter assembly protein HypD | D6M20_12425 |
|  | [NiFe] hydrogenase metallocenter assembly protein HypC | D6M20_12430 |
|  | [NiFe] hydrogenase metallocenter assembly protein HypF | D6M20_12435 |
|  | Hydrogenase assembly chaperone HypC/HupF | D6M20_12440 |
|  | Hydrogenase maturation protease | D6M20_12445 |
| **Degradation of hydrocarbons and organic compounds** | Alkane-1 monooxygenase (alkB) | D6M20_00720 (plasmid), D6M20_13355, D6M20_14305, D6M20_18855, D6M20_21045, D6M20_27785 |
|  | Catechol 1,2-dioxygenase (catA) | D6M20_27935 |
|  | Dibenzothiophene desulfurization enzyme B (dszB ) | D6M20_25605 |
| **Glucosinolate metabolism** | branched-chain amino acid aminotransferase | D6M20_08100 |
|  | 3-isopropylmalate/(R)-2-methylmalate dehydratase large subunit | D6M20_13320 |
|  | 3-isopropylmalate dehydratase small subunit (EC 4.2.1.33) | D6M20_13315 |
|  | ß-glucosidase (myrosinase) | D6M20_11475 |
|  | Peptide-methionine (S)-S-oxide reductase/methionine sulfoxide reductase A *msrA* | D6M20_23985 |
|  | Peptide-methionine (S)-S-oxide reductase/mehionine sulfoxide reductase B *msrB* | D6M20_11510 |
|  | aldoxime dehydratase *oxd* | D6M20_25300 |
|  | Alkyl sulfatase and related hydrolases, MBL-fold metallo-hydrolase superfamily | D6M20_19070 |
|  | cobalt-containing nitrile hydratase subunit beta | D6M20_25275 |
|  | cobalt-containing nitrile hydratase subunit alpha | D6M20_25280 |
|  | amidase clustered with urea ABC transporter and nitrile hydratase functions | D6M20_25285 |
|  | formamidase | D6M20_17170 |
|  | 5-aminopentanamidase | D6M20_21895 |
| **Bile acid degradation** | Bile acid 7-alpha dehydratase BaiE | D6M20_04375 |
| **Indole acetic acid production** | tryptophan synthase alpha chain | D6M20_09210 |
|  | tryptophan synthase beta chain | D6M20_09205 |
|  | anthranilate phosphoribosyltransferase | D6M20_08200 |
|  | Indole-3-glycerol phosphate synthase | D6M20_09200 |
|  | amidase *amiE* | D6M20_30045 |
|  | amine oxidase *iaaM* (identified as iaaM in PIFAR, blastP 56% identity with Psyr_1536) | D6M20_13780 |
| **Salicylic acid** | salicylate hydroxylase | D6M20_24110 |
| **Gibberelin production** | SDR family oxidoreductase | D6M20_09080 |
|  | Lanosterol 14-alpha demethylase @ Cytochrome P450 51 | D6M20_09085 |
|  | ferredoxin | D6M20_09090 |
|  | Geranylgeranyl diphosphate synthase | D6M20_08310 |
| **Cytokinin** | Phosphoribohydrolase (homolog of plant cytokinin-activating enzyme LOG) | D6M20_05240 |
| **Glutamate biosynthesis** | glutamate synthase large subunit *gltB* | D6M20_09230, D6M20_24575 |
|  | glutamate synthase small subunit | D6M20_09235, D6M20_24570 |
| **Spermidine** | arginine decarboxylase SpeA | D6M20_01150 (plasmid) |
|  | agmtinase (agmatine deiminase) SpeB | D6M20_03950 |
|  | spermidine synthase SpeE | D6M20_07935, D6M20_29000 |
| **Siderophore production** | chorsimate synthase | D6M20_10990 |
|  | class I SAM-dependent methyltransferase | D6M20_12200 |
|  | amino acid adenylation domain-containing protein | D6M20_12245 |
|  | isochorismatase | D6M20_12255 |
|  | 2,3-dihydroxybenzoate-AMP ligase | D6M20_12260 |
|  | 2,3-dihydro-2,3-dihydroxybenzoate dehydrogenase | D6M20_12265 |
|  | isochorismatase | D6M20_12270 |
|  | isochorismatase synthase | D6M20_12275 |
|  | ABC transporter ATP-binding protein | D6M20_12210 |
|  | MFS transporter | D6M20_12225 |
|  | enterobactin transporter EntS | D6M20_12235 |
|  | ABC transporter substrate-binding protein | D6M20_12240 |
|  | ATP-binding cassette domain-containing protein | D6M20_12305 |
|  | sigma-70 family RNA polymerase sigma factor | D6M20_12190 |
|  | TipAS antibiotic-recognition domain-containing protein | D6M20_12335 |
|  | TetR/AcrR family transcriptional regulator | D6M20_12350 |
|  | o-succinylbenzoate synthase | D6M20_20205 |
|  | M20 family metallopeptidase | D6M20_20215 |
|  | MbtH family protein | D6M20_20290 |
|  | lysine N(6)-hydroxylase/L-ornithine N(5)-oxygenase family protein | D6M20_20295 |
|  | non-ribosomal peptide synthetase | D6M20_20300 |
|  | MMPL family transporter | D6M20_20310 |
|  | alpha/beta hydrolase | D6M20_20325 |
|  | methionyl-tRNA formyltransferase | D6M20_20330 |
|  | MFS transporter | D6M20_20225 |
|  | ATP-binding cassette domain-containing protein | D6M20_20335 |
|  | ABC transporter ATP-binding protein | D6M20_20340 |
|  | HAMP domain-containing histidine kinase | D6M20_20245 |
|  | response regulator transcription factor | D6M20_20250 |
| **Iron acquisition** | ferrous iron transport peroxidase EfeB | D6M20_04645 |
|  | ferrous iron transport permease EfeU | D6M20_04655 |
|  | ferrous iron transport periplasmic protein EfeO | D6M20_04650 |
| **Organic acid production** | 6-phosphogluconate dehydrogenase | D6M20_10425, D6M20_24825 |
|  | L-lactate dehydrogenase | D6M20_05005 |
| **Phosphate metabolism** | alkaline phosphatase | D6M20_15135 |
|  | inorganic pyrophosphatase | D6M20_21710 |
|  | Phosphate ABC transporter, periplasmic phosphate-binding protein PstS | D6M20_30820 |
|  | Phosphate transport system permease protein PstC | D6M20_30825 |
|  | Phosphate transport system permease protein PstA | D6M20_30830 |
|  | Phosphate transport ATP-binding protein PstB | D6M20_30835 |
| **Aromatic carbon metabolism** | enoyl-CoA hydratase | D6M20_19280 |
|  | fumarylacetoacetate hydrolase | D6M20_19325 |
| **Nitrogen metabolism** | uncharacterized NifU-like protein (MSMEG_2718) | D6M20_12465 |
|  | Respiratory nitrate reductase gamma chain (EC 1.7.99.4) | D6M20_00810 |
|  | Respiratory nitrate reductase delta chain (EC 1.7.99.4), nitrate reductase molybdenum cofactor assembly chaperone | D6M20_00815 |
|  | Respiratory nitrate reductase beta chain (EC 1.7.99.4) | D6M20_00820 |
|  | Respiratory nitrate reductase alpha chain (EC 1.7.99.4) | D6M20_00825 |
|  | FdhF/YdeP family oxidoreductase | D6M20_00830 |
| **Carbon metabolism** | LacI transcription regulator | D6M20_14825, D6M20_21260, D6M20_30610 |
|  | aldo-keto reductase | D6M20_03265, D6M20_03280, D6M20_05680, D6M20_09925, D6M20_13825, D6M20_14655, D6M20_29790 |
| **Volatiles** | acetolactate synthase small subunit *budB* | D6M20_13380 |
|  | acetolactate synthase large subunit *budB* | D6M20_13385 |
|  | (R,R)-butanediol dehydrogenase | D6M20_05945, D6M20_29895 |
|  | lipoyl synthase *lipA* | D6M20_08065, D6M20_29915 |
| **Exopolysaccharides** | UTP--glucose-1-phosphate uridylyltransferase *galU* | D6M20_04080, D6M20_18535 |
| **Proteases** | periplasmic serine endoprotease DegP-like *htrA* | D6M20_05305 |
| **Biofilm formation** | phosphoglucomutase | D6M20_13745 |
|  | signal peptidase I W | D6M20_27405, D6M20_13075, D6M20_17775 |
| **MAMP** | chemotaxis protein CheY | D6M20_26160 |
| **Antibiotic production** | dTDP-glucose 4,6-dehydratase | D6M20_23600 |
| **Quorum sensing** | two-component transcriptional response regulator from the LuxR family | D6M20_29615 |
| **Quorum quenching** | *qsdA* Aryldialkylphosphatase (phosphotriesterase/paraoxonase/putative php); putative N-Acyl homoserine lactonase | D6M20_27580 |

## 2. Supplementary Figures

##
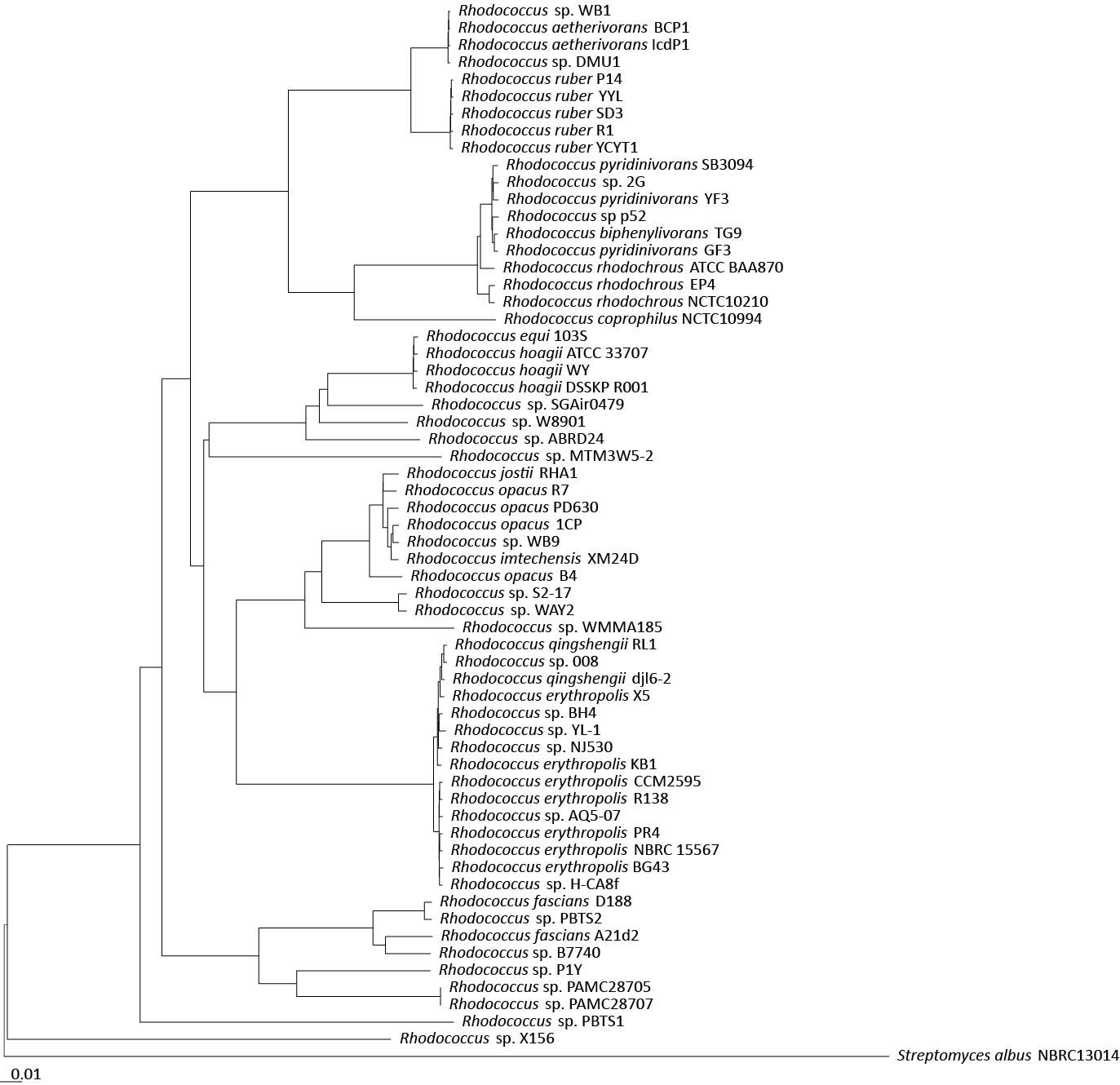


**Figure S1**: Maximum-likelihood phylogenetic tree of the *Rhodococcus* genus generated with FastTree from nucleotide sequences of the core genome. Values represent local support values based on Shimodaira-Hasegawa test (1 SH = 100% bootstrap). The scale bar represents nucleotide substitutions per site (0.01 scale = 1% nucleotide substitutions per site).

##
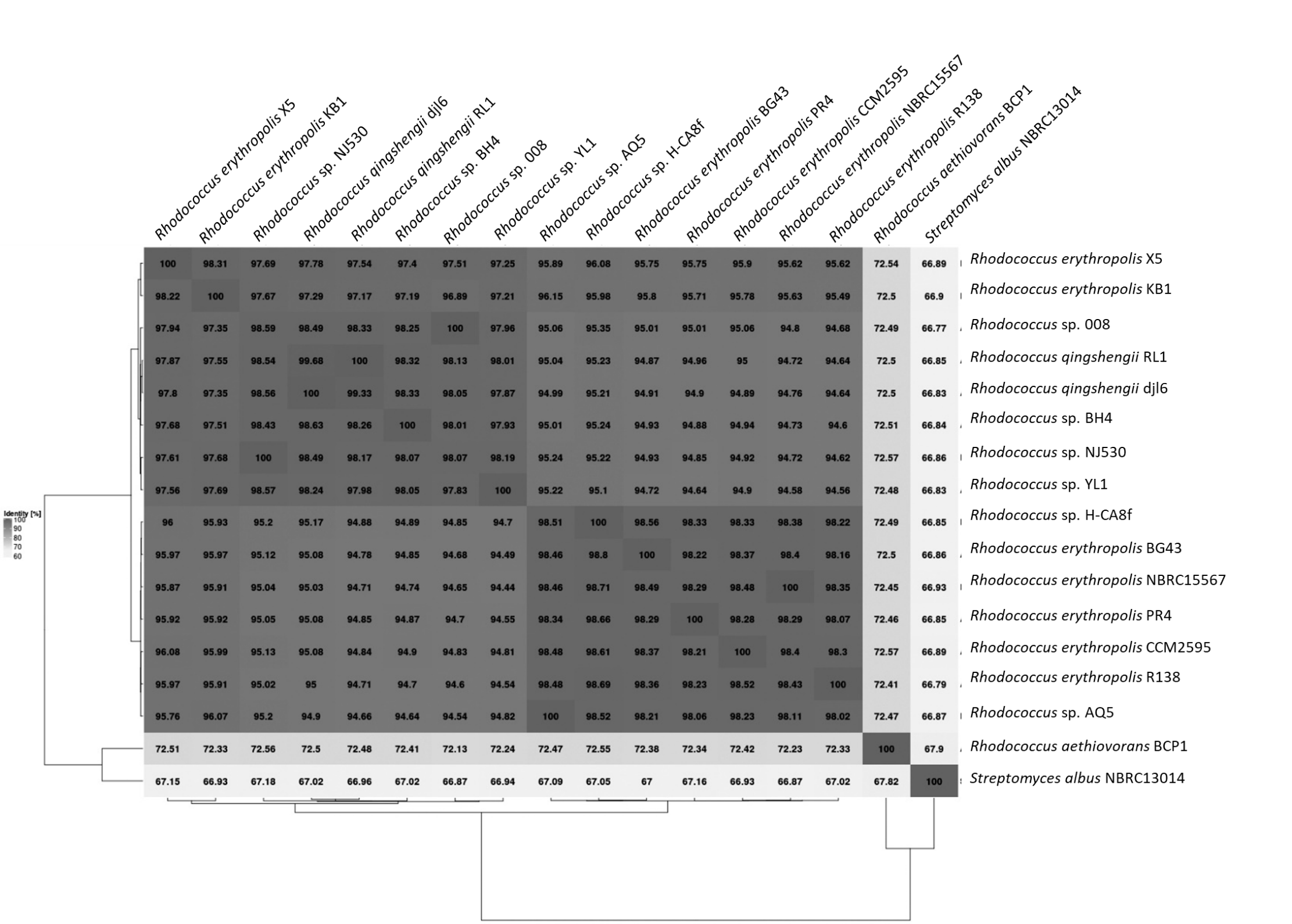


**Supplementary Figure S2:** Average Nucleotide Identity (ANI) Matrix of *Rhodococcus erythropolis* clade based on 15 full *Rhodococcus* genomes + *Rhodococcus aethiovorans* BCP1 and *Streptomyces alba* NBRC13014 as outgroups. Data obtained with EDGAR Software.


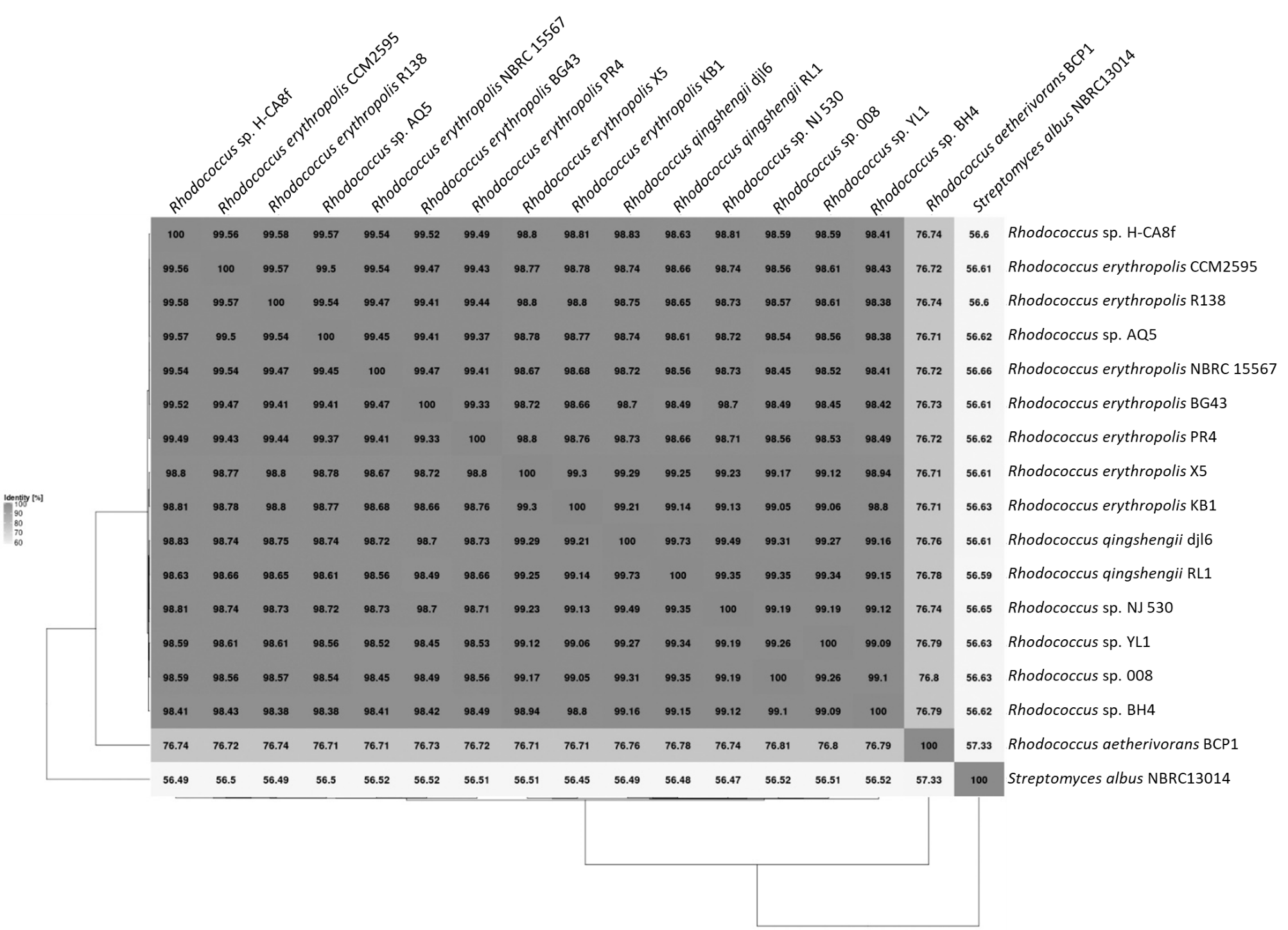


**Figure S3**: Average Aminoacid Identity (AAI) Matrix of *Rhodococcus erythropolis clade* based on 15 full genomes + *Rhodococcus aethiovorans* BCP1 and *Streptomyces alba* NBRC13014 as outgroups. Data obtained with EDGAR Software.


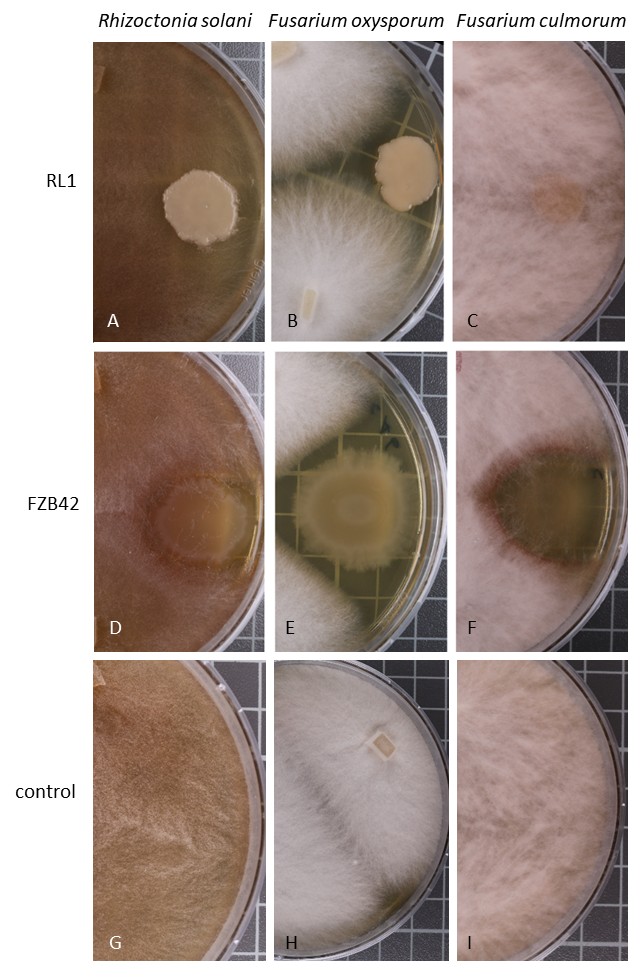


**Figure S4**: Confrontation assay of RL1 (**A-C**) and the positive control FZB42 (**D-F**) against plant pathogenic fungi after 9 days of plate cultivation. Antagonistic activity is indicated by inhibition zone around bacteria. Control plates (**G-I**) without bacteria
